# Design and synthesis of a tetraphenylethene-porphyrin hetero-faced molecular cage for photodynamic therapy

**DOI:** 10.1093/nsr/nwag241

**Published:** 2026-04-22

**Authors:** Jiaxi Fan, Zhuoxia Li, Lin Cheng, Zhaolong Wang, Zhihui Guo, Pingxia Wang, Didi Chen, Jiao Zhu, Yuting Wang, Jun Li, Xianglong Duan, Min Li, Liping Cao

**Affiliations:** College of Chemistry and Materials Science, Northwest University, Xi’an 710069, China; Department of Hepatobiliary Surgery, Union Hospital, Tongji Medical College, Huazhong University of Science and Technology, Wuhan 430022, China; College of Chemistry and Materials Science, Northwest University, Xi’an 710069, China; State Key Laboratory of Chemical Reaction Dynamics, Dalian Institute of Chemical Physics, Chinese Academy of Sciences, Dalian 116023, China; College of Chemistry and Materials Science, Northwest University, Xi’an 710069, China; College of Chemistry and Materials Science, Northwest University, Xi’an 710069, China; Hubei Key Laboratory of Purification and Application of Plant Anti-Cancer Active Ingredients, Hubei University of Education, Wuhan 430205, China; Department of Gastroenterology, The First Affiliated Hospital of Xi’an Jiaotong University, Xi’an 710061, China; Shaanxi Engineering Research Center of Medical Polymer Materials, Second Department of General Surgery, Shaanxi Provincial People’s Hospital, Xi’an 710068, China; College of Chemistry and Materials Science, Northwest University, Xi’an 710069, China; Shaanxi Engineering Research Center of Medical Polymer Materials, Second Department of General Surgery, Shaanxi Provincial People’s Hospital, Xi’an 710068, China; Shaanxi International Science and Technology Cooperation Base for Clinical Medicine, Xi’an 710068, China; School of Life Science and Technology, Northwestern Polytechnical University, Xi’an 710072, China; Second Department of General Surgery, Third Affiliated Hospital of Xi’an Jiaotong University, Xi’an 710068, China; Department of Hepatobiliary Surgery, Union Hospital, Tongji Medical College, Huazhong University of Science and Technology, Wuhan 430022, China; College of Chemistry and Materials Science, Northwest University, Xi’an 710069, China

**Keywords:** supramolecular chemistry, molecular cage, tetraphenylethene, porphyrin, photodynamic therapy

## Abstract

The development of efficient photosensitizers for photodynamic therapy (PDT) remains a significant challenge due to the limitations of aggregation-caused quenching (ACQ) in commonly used chromophores. Here, we present the design and synthesis of a tetraphenylethene-porphyrin hetero-faced molecular cage (**1•**8Clˉ), where tetraphenylethene (TPE) with aggregation-induced emission (AIE) properties is covalently linked to porphyrin, which exhibits good photosensitivity but suffers from ACQ effects. The hetero-faced molecular cage is designed with a face-to-face configuration, facilitated by four *p-*xylylene linkers, ensuring precise spatial alignment of the TPE and porphyrin units. This cage-type molecular architecture not only enables the conversion of ACQ to AIE, but also populates the triplet state of porphyrin via efficient intramolecular energy and electron transfer owing to the favorable geometry. As a result, **1•**8Clˉ demonstrates excellent ability to generate reactive oxygen species (ROS) and binds nicotinamide adenine dinucleotide (NADH) in aqueous solution, catalyzing the rapid photocatalytic oxidation of NADH to its oxide form (NAD^+^). Utilizing ROS generation and the disruption of the intracellular redox balance of NADH, **1•**8Clˉ exhibits significant potential for effective photocatalysis-assisted PDT in hypoxic tumor environments. This study opens a new pathway for molecular design by combining molecular cage structures with photosensitizer functionality, enabling applications in fields such as photocatalysis and PDT.

## Teaser

The tetraphenylethene-porphyrin hetero-faced molecular cage facilitates intramolecular energy and electron transfer, effectively generates ROS and promotes photocatalytic oxidation of NADH, demonstrating advanced potential for photodynamic therapy in hypoxic tumors.

## INTRODUCTION

Photofunctional macrocyclic compounds have recently attracted considerable attention due to their potential applications in molecular recognition, sensing, catalysis, and biomedicine [1]. Particularly, the incorporation of chromophores into the macrocyclic structure enhances the system’s photophysical properties [2]. Compared to single-chromophore systems, the incorporation of multiple chromophores enhances the efficiency and tunability of light-emitting or light-harvesting properties, owing to the precise control over chromophore types, relative ratios, and spatial configuration [3,4]. The precise configuration of heterogeneous chromophores in the macrocyclic structure offers an effective strategy for regulating interchromophoric interactions and photophysical processes, such as energy transfer (EnT) and/or electron transfer (ET), as demonstrated in a limited number of reported hetero-faced cyclophanes [5–9]. The designable three-dimensional (3D) macrocyclic structure of molecular cage, with the chromophores as the cage’s faces arranged in a face-to-face configuration, can serve as an ideal platform for constructing such multichromophoric systems, facilitating the integration of diverse photofunctional building blocks [10–13]. Moreover, the 3D inherent cavity of molecular cages can encapsulate guest molecules to further modulate its functional properties, offering an innovative and efficient approach for developing advanced photofunctional supramolecular systems [14–16].

Porphyrins and their derivatives are key natural chromophores [17,18], and have been widely utilized in optoelectronic materials [19,20], photocatalysis [21,22], and photodynamic therapy (PDT) [23–26], owing to their visible-to-near-infrared emission, excellent light-harvesting capabilities, redox properties, and photochemical stability. However, their extended coplanar π-conjugated structures facilitate strong intermolecular π···π interactions that result in stacked packing, often causing aggregation-caused quenching (ACQ) of fluorescence in aqueous solution. In recent years, aggregation-induced emission (AIE), which is opposite to the ACQ effect, has garnered significant attention due to its unique fluorescent emission ability in the aggregated state, providing great potential for addressing the ACQ issue [27,28]. Typically, tetraphenylethylene (TPE), an AIE fluorophore, shows enhanced fluorescence in the aggregated state, overcoming the ACQ effect [29,30]. Integrating ACQ and AIE chromophores through covalent bonds provides a promising strategy to overcome ACQ limitations and design high-performance multichromophoric fluorescent systems [31,32].

**Figure 1.**
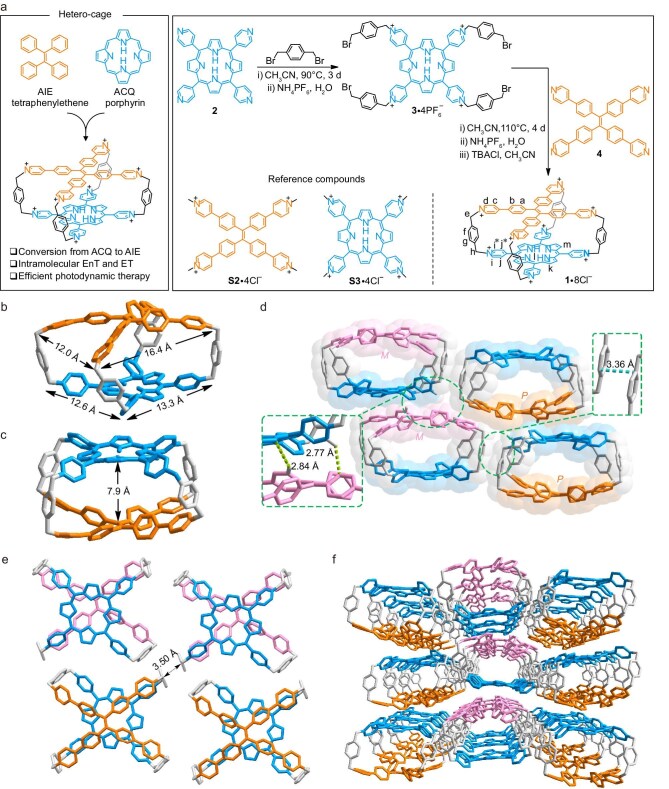
(a) Synthetic route of **1•**8Clˉ and chemical structures of **S2•**4Clˉ and **S3•**4Clˉ. X-ray structures of **1•**8Clˉ: (b) diagonal view, (c) side view, stack packing from (d) *c*-axis and (e) *b*-axis, and (f) perspective view of the 3D framework from *a*-axis. For clarity, all hydrogen atoms, counterions, and solvent molecules are omitted.

Although porphyrin and TPE units have been individually employed as molecular building blocks to construct homo-faced molecular cages, no synthetic studies have yet been reported that combine both molecular modules within the same cage structure [33–40]. The synthesis of this hetero-faced molecular cage is challenging due to the size mismatch between the two molecular modules. This discrepancy increases the molecular strain generated during multiple-site cyclization, leading to lower yields and greater difficulty in separation and purification. Here, we address this challenge by presenting the design and synthesis of a TPE-porphyrin hetero-faced molecular cage (**1•**8Clˉ), which incorporates both porphyrin and TPE units (Fig. 1a). In the molecular design strategy, TPE, which possesses AIE characteristics, covalently links with porphyrin units known for their excellent photosensitivity but prone to ACQ effects. This cage-type architecture successfully converts the ACQ behavior of porphyrin into AIE properties. Specifically, the molecular cage adopts a distinctive face-to-face configuration between the TPE and porphyrin units, which effectively shortens the spatial distance between these two moieties. This proximity facilitates efficient intramolecular EnT and ET pathways to populate the triplet state of porphyrin, enhancing the generation efficiency of reactive oxygen species (ROS). Beyond improving optical performance, the 3D hydrophobic cavity within the cage structure enables aqueous molecular recognition of nicotinamide adenine dinucleotide (NADH) by forming 1:2 host–guest complex. This molecular recognition promotes the photocatalytic oxidation of NADH to its oxide form (NAD^+^), thereby disrupting the biochemical equilibrium of this biomolecule within cancer cells. Importantly, this system demonstrates effective PDT in hypoxic tumor environments. The unique design of the molecular cage, by the combination of TPE chromophore and porphyrin photosensitizer, can efficiently damage the cancerous microenvironment and induce cell death.

## RESULTS AND DISCUSSION

### Synthesis and structural analysis

To integrate two different chromophores into a cage-type architecture, we adopted a two-step reaction approach to synthesize the hetero-faced molecular cage. Indeed, we explored two synthetic pathways. The first one involved a cyclization reaction between a TPE-based intermediate (**S1•**4PF_6_ˉ), which had previously been successfully used in the synthesis of a TPE-based homo-faced molecular cage [34], and tetrapyridyl porphyrin (**2**). However, this synthetic pathway yielded extremely trace amounts of **1•**8PF_6_ˉ, with a yield of 0.32% (synthetic route in supporting information). We hypothesize that the size of the tetrapyridyl TPE face in **S1•**4PF_6_ˉ is larger than that in compound **2**. This increased size prevents the four benzylic bromide groups in **S1•**4PF_6_ˉ from overcoming the molecular strain needed to react with the four pyridyl groups in compound **2**, rendering this approach an unviable synthetic method. Subsequently, we adopted another synthetic route. As shown in Fig. 1a, **1•**8Clˉ was successfully synthesized via two-step S_N_2 reactions. The porphyrin-based intermediate (**3•**4PF_6_ˉ) was obtained by reacting compound **2** with an excess of 1,4-bis(bromomethyl)benzene in CH_3_CN at 90°C for 3 days, affording a yield of ∼65%. At this point, the size of the porphyrin face in **3** is smaller than that of the tetrapyridyl TPE (**4**), where the molecular strain in the four benzylic bromide groups of **3** is smaller, allowing them to successfully react with the four pyridyl groups of **4**. As a result, the formation of **1•**8PF_6_ˉ was carried out in CH_3_CN at 110°C using an equimolar mixture of **3•**4PF_6_ˉ and **4** in ∼5.3% yield after purification by column chromatography. The water-soluble form (**1•**8Clˉ) was obtained from **1•**8PF_6_ˉ by counterion exchange in ∼92% yield. The chemical structures of all compounds were confirmed by ^1^H, ^13^C, COSY, and NOESY nuclear magnetic resonance (NMR) spectroscopy, as well as electrospray ionization mass spectrometry (ESI-MS) (Figs S1–S10). Additionally, tetra(*N-*methylpyridinium) TPE (**S2•**4Clˉ) and tetra(*N-*methylpyridinium) porphyrin (**S3•**4Clˉ), as reference monomers, were synthesized based on the reported synthetic route (Fig. 1a).

Solid evidence for the formation of the hetero-faced cage is provided by single-crystal X-ray diffraction (Table S1). Dark red single crystals of **1•**8Clˉ were obtained by vapor diffusion of *i-*Pr_2_O into a CH_3_OH solution of **1•**8Clˉ at room temperature. In the X-ray structure, **1** adopts a cage-like asymmetric geometry resembling a frustum shape. The sizes of the porphyrin-based face are ≈12.6 Å × 13.3 Å, while those of the TPE-based face are ≈12.0 Å × 16.4 Å (Fig. 1b). The centroid-to-centroid distance between the two faces is ∼7.9 Å (Fig. 1c), which is optimal for the molecular cage as a host to selectively recognize aromatic groups through π···π interactions. In contrast to the nearly coplanar conformation of the pyridinium cation ring and the benzene ring of the TPE core in the TPE face, the pyridinium cation ring in the porphyrin face forms dihedral angles (≈46.1°–52.8°) with the porphyrin plane, resulting in a non-coplanar conformation. This conformational feature, due to its slow exchange rate, can be distinguished by NMR at 298 K, where the proton resonances of the pyridinium cation are split into two sets (Figs S3 and S5): one set of hydrogen atoms (H_i_–H_j_) points outward from the cavity, while the other set points inward toward the cavity (H_i_*–H_j_*). In the crystal, the molecular cage adopts two chiral conformations, displaying a mirror-symmetric structure. The entire crystal is racemic, with TPE-based right-handed (*P*) and left-handed (*M*) rotational conformations. Notably, the stacking structure between these chiral conformational isomers features an alternating arrangement between *P-*/*M-*rotational TPE and porphyrin units, which disrupts the self-aggregation typically observed for porphyrin moieties. And the packing is further stabilized by multiple weak interactions, including C–H···π interactions (∼2.8 Å) and π···π interactions (∼3.4 Å) between the TPE-based unit and the porphyrin-based unit in another adjacent cage (Fig. 1d and Fig. S11). The *p-*xylylene rings in the closest adjacent cages exhibit intermolecular π···π interactions with an average distance of ∼3.5 Å to achieve a 3D stack packing (Fig. 1e). Furthermore, a 3D nanotubular framework is observed in Fig. 1f, which features intrinsic nanotubes constructed by the cavities of **1•**8Clˉ along the *a*-axis. This type of packing suggests that the cage-type architecture, due to the steric effects of its 3D spatial structure, effectively prevents π···π stacking between the porphyrin rings, potentially eliminating the ACQ effect.

### Photophysical properties

**Figure 2.**
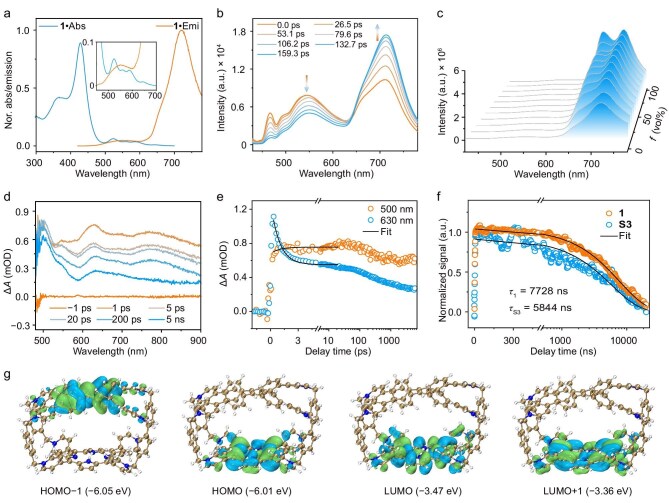
(a) Normalized ultraviolet-visible (UV-vis) absorption (Nor. abs) and fluorescence spectra of **1•**8Clˉ (10 μM) in water (Abbreviations: Abs, absorption; Emi, emission). (b) Time-resolved fluorescence spectra of **1•**8Clˉ (10 μM, λ_ex_ = 400 nm) in water. (c) Fluorescence spectra of **1•**8Clˉ (10 μM) in the CH_3_CN-H_2_O mixture. The *f* (vol%) represents the volume percentage of acetonitrile in the mixed solution. (d) The fs-TA spectra and (e) kinetic curves of **1•**8Clˉ (10 μM, λ_ex_ = 400 nm) in water. (f) The ns-TA kinetic curves of **1•**8Clˉ (λ_ex_ = 400 nm) and **S3•**4Clˉ (λ_ex_ = 530 nm) in water. (g) The HOMO–LUMO distributions and calculated energy levels of **1**.

Due to the presence of both TPE and porphyrin chromophores within a single cage-type structure, we speculate that the EnT and/or ET could occur between these two different chromophores with complementary photophysical properties, facilitated by their spatial proximity (∼7.9 Å) in the face-to-face configuration. And its photophysical properties were investigated. The steady-state absorption and emission spectra of **1•**8Clˉ in water are shown in Fig. 2a. The absorption spectrum of **1•**8Clˉ features a prominent Soret band at λ_max_ = 428 nm, alongside four weaker Q bands within the 500–700 nm range, which are the characteristic of the porphyrin unit. Additionally, a broad absorption band around ≈300–450 nm is observed, which is attributed to the TPE unit. These results suggest that, despite the covalent linkage of both TPE and porphyrin chromophores within the same molecular cage, the ground-state absorption spectra of the two chromophores remain unaffected by mutual interactions, demonstrating a simple additive superposition of their individual absorption characteristics. However, under excitation conditions, the molecular cage exhibits intramolecular EnT behavior between the TPE and porphyrin subcomponents. The fluorescence emission spectrum of **1•**8Clˉ exhibits two distinct peaks at λ_max_ = 667 and 720 nm, arising from the porphyrin unit. The TPE unit only displays a weak, broad emission band with a maximum at λ_max_ = 542 nm, which overlaps with the Q-band absorption of the porphyrin unit (Fig. 2a, inset). These results indicate the potential intramolecular fluorescence resonance energy transfer from the TPE to the porphyrin units within **1•**8Clˉ. The time-resolved emission spectra (TRES) of **1•**8Clˉ clearly showed that the emission of the TPE unit decayed, while the emission intensity of the porphyrin unit increases over the subsequent 159.3 ps, which supports an intramolecular EnT process from the TPE to the porphyrin units (Fig. 2b and Fig. S12). Additionally, the TRES of the non-covalent mixed system of **S2•**4Clˉ and **S3•**4Clˉ, as reference monomers, only exhibit the combined spectral features of the two chromophores, with no evidence of an EnT process (Figs S13 and S14), further confirming that efficient EnT processes occur exclusively within the cage structure. Within the molecular cage, the fluorescence lifetime of the TPE unit increased to 4.3 ns at 550 nm, compared to the TPE monomer **S2•**4Clˉ (2.08 ns) (Table S2). This change may be attributed to the structural confinement within the molecular cage, which restricts the free rotation of TPE, thereby enhancing the stability of its excited state. In contrast, the fluorescence lifetime of the porphyrin unit decreased to 3.78 ns at 700 nm, compared to the porphyrin monomer **S3•**4Clˉ (5.48 ns). The reduced fluorescence lifetime of the porphyrin unit may result from competition with other photophysical processes (for example, electron transfer), which hinder its radiative transition pathway. The AIE properties of **1•**8Clˉ were further investigated in a CH_3_CN/H_2_O solvent mixture. The emission intensity of **1•**8Clˉ gradually increases as the CH_3_CN fraction is raised from 0% to 99% in the CH_3_CN/H_2_O mixture (Fig. 2c). These observations suggest that the conjugation between the TPE and the porphyrin units likely inhibits π···π stacking of the porphyrin moieties, thereby promoting porphyrin emission via the intramolecular EnT process from the TPE unit, and facilitating the conversion of the entire covalent molecular system from ACQ to AIE properties.

To investigate the excited-state properties of **1•**8Clˉ, particularly regarding its intramolecular EnT and ET between the TPE and porphyrin units, we employed femtosecond and nanosecond transient absorption (fs-TA and ns-TA, respectively) spectroscopy on **1•**8Clˉ as the target compound, with **S2•**4Clˉ and **S3•**4Clˉ serving as references. Upon photoexcitation at 400 nm, both **1•**8Clˉ and **S2•**4Clˉ exhibit positive features at ca. 630 nm, which can be attributed to the excited-state absorption (ESA) of the singlet excited states of TPE unit (Fig. 2d and Fig. S15). Under excitation at 530 nm, which is the wavelength that can directly excite the porphyrin unit, **S3•**4Clˉ shows weak positive features at ca. 500, 550, and 610 nm, that persist over the whole-time window (Fig. S16). Such a long-lived excited species can be attributed to the triplet excited states of porphyrin stemming from intersystem crossing (ISC). Similar spectral features were observed in **1•**8Clˉ at the same timescale, in which, positive features at ca. 500 is assigned to the ESA of the triplet excited states of the porphyrin unit (T_1_→T_n_). On the early time window, we observed the rapid ESA signal decay of the singlet excited states of the TPE unit and concomitant emergence of the ESA signal of the porphyrin unit, which is an indicative of EnT from the excited TPE to the porphyrin unit (Fig. 2e). This process is strongly supported by the TRES (Fig. 2b). The kinetic curves probed at 500 and 630 nm are simultaneously exponential fitted, giving ∼0.9 ps as the time constant of the EnT. Notably, ns-TA experiments show that the lifetime of the formed triplet excited states of porphyrin in **1•**8Clˉ is effectively extended by 1.3-fold than the control **S3•**4Clˉ (Fig. 2f and Figs S17–S18). With excitation at 400 nm, therefore, the nascent singlet excited-state TPE unit in **1•**8Clˉ undergoes fast intramolecular EnT to the ground-state porphyrin unit, forming the singlet excited-state porphyrin unit, which subsequently undergoes ISC to the long-lived triplet excited states.

In addition, by comparing the steady-state fluorescence spectra of **S3•**4Clˉ, the 1:1 mixture of **S2•**4Clˉ and **S3•**4Clˉ, and **1•**8Clˉ under 530 nm excitation, a significant decrease in the fluorescence intensity of **1•**8Clˉ was observed, suggesting the possibility of intramolecular ET within the molecular cage upon the selective excitation of the porphyrin unit in **1•**8Clˉ (Fig. S19). The fs-TA spectra of **1•**8Clˉ collected under same excitation wavelength reveals the appearance of absorption characteristic peaks at 500 and 650 nm, corresponding to the porphyrin^•−^ due to the charge-separation (Fig. S20) [41]. These peaks indicate that **1•**8Clˉ undergo a rapid ET from TPE to porphyrin after excitation. It is found that the spectral features from 500 ps to 5 ns are consistent with the one observed under the 400 nm excitation condition, indicating the formation of the triplet state of porphyrin. The cyclic voltammetry curve indicates that **S2•**4Clˉ has a more negative first reduction potential (−1.037 V vs. −0.535 V for **S3•**4Clˉ, using Ag/AgCl as the reference electrode), suggesting that the lowest unoccupied molecular orbital (LUMO) energy level of **S2•**4Clˉ is higher than that of **S3•**4Clˉ (Fig. S21 and Table S3). This LUMO level alignment suggests that photoinduced ET from **S2•**4Clˉ to **S3•**4Clˉ is thermodynamically favorable. Moreover, distinct negative shifts in both the first oxidation and reduction potentials of **1•**8Clˉ compared to the monomers of TPE and porphyrin units, indicating the possible electrons communication generated by the face-to-face configuration of the TPE and porphyrin units in **1•**8Clˉ. Furthermore, density functional theory (DFT) calculations based on the optimized geometry were performed to gain insight into the energies and electron orbitals. The results show that the highest occupied molecular orbital (HOMO-1) of **1** is completely distributed over the TPE unit, while HOMO and the LUMO and LUMO + 1 is localized on the porphyrin moiety, which further confirms that the HOMO-to-LUMO transition possesses a possibility of the intramolecular ET from the TPE to the porphyrin units (Fig. 2g). The calculated electronic orbitals indicate that the spatial separation achieved through the structure of the molecular cage creates a physical distance between TPE and porphyrin that facilitates photo-induced charge separation and recombination to the triplet state [42,43]. The time-dependent DFT (TD-DFT) calculations reveal that **1•**8Clˉ has a small energy gap (Δ*E*_ST_) between the singlet state and the triplet state of ∼0.10 eV (S_1_–T_4_) between the lowest single and triple excited states, facilitating efficient ISC (Fig. S22). Taken together, these results suggest that both intramolecular EnT and ET processes within the molecular cage promote the generation of the triplet state, which benefit from the favorable geometry of the cage [44,45].

### ROS generation

**Figure 3.**
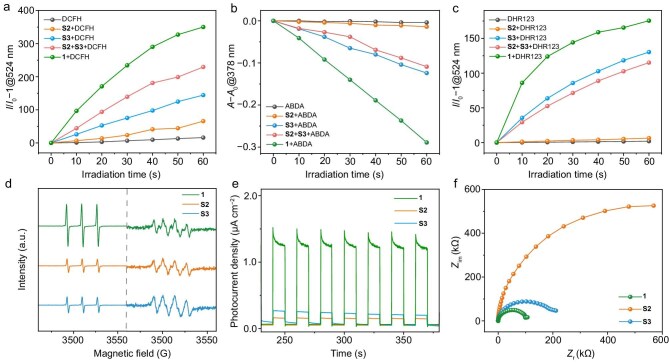
Plots for (a) ROS, (b) ^1^O_2_, and (c) O_2_^•−^ generation of **1**•8Clˉ, **S2**•4Clˉ, **S3**•4Clˉ, and the 1:1 mixture of **S2**•4Clˉ and **S3**•4Clˉunder irradiation (white LED lamp, 20 mW cm^−2^) by using DCFH, ABDA, and DHR123 as the indicators, respectively. (d) EPR spectra for ^1^O_2_ and O_2_^•−^ generated from **1**•8Clˉ, **S2**•4Clˉ, and **S3**•4Clˉwith white-light irradiation (Xe lamp, 300 W) for 30 s, using TEMP and DMPO as spin trap agents, respectively. (e) Representative photocurrent responses of **1**•8Clˉ, **S2**•4Clˉ, and **S3**•4Clˉ on a carbon paper electrode with the interval of 10 s, respectively. (f) Charge transfer resistance of **1**•8Clˉ, **S2**•4Clˉ, and **S3**•4Clˉ under white-light irradiation (480 mW cm^−2^), respectively.

Due to the efficient intramolecular EnT and ET processes of **1•**8Clˉ, which can be induced by visible light (for example, 400 and 530 nm, respectively), the molecular cage exhibits an extended triplet-state lifetime. The photosensitizing capability of **1•**8Clˉ for ROS generation was evaluated under white-light irradiation (20 mW cm^−2^), with **S2•**4Clˉ, **S3•**4Clˉ and the 1:1 mixture of **S2•**4Clˉ and **S3•**4Clˉ as the control groups. First, 2′,7′-dichlorodihydrofluorescein (DCFH) was employed as a general ROS indicator to evaluate the ROS-generating ability of these four groups of samples in water (Fig. 3a and Fig. S23). Upon irradiation in the presence of **1•**8Clˉ, a significantly enhanced green fluorescence emission of DCFH at λ_max_ = 524 nm was observed, indicating substantial ROS generation by **1•**8Clˉ. The fluorescence enhancement of control groups was much smaller than that of **1•**8Clˉ, demonstrating that **1•**8Clˉ exhibited the highest total ROS production capacity. Next, 9,10-anthradiyl-bis(methylene) dimalonic acid (ABDA) undergoes a decrease in absorbance upon reaction with singlet oxygen (^1^O_2_), making it a reliable probe for the detection of Type-II ROS. Under white-light irradiation, compared to the control groups, the addition of **1•**8Clˉ induced faster decomposition of ABDA, accompanied by a significant reduction in the absorbance at 378 nm, indicating a faster and more efficient ^1^O_2_ generation by **1** (Fig. 3b and Fig. S24). Then, dihydrorhodamine 123 (DHR123), a specific free radical indicator of Type-I ROS, was selected for real-time monitoring of superoxide anion (O_2_^•−^) generation. The most significant fluorescence enhancement at λ_max_ = 525 nm was observed in the DHR123 solution containing **1•**8Clˉ compared to the control groups under white light illumination, confirming the effective production of O_2_^•−^ by **1•**8Clˉ (Fig. 3c and Fig. S25). Electron paramagnetic resonance (EPR) measurements using 2,2,6,6-tetramethylpiperidine (TEMP) and 5,5-dimethyl-1-pyrroline-*N-*oxide (DMPO) as spin-trapping agents for ¹O_2_ and O_2_^•−^, respectively, further validated the generation of these ROS species by **1•**8Clˉ (Fig. 3d and Figs S26–S27). The experimental evidence using these ROS scavengers demonstrated that **1•**8Clˉ exhibits excellent ROS generation under irradiation conditions, including ^1^O_2_ and O_2_^•−^. Photocurrent tests indicated **1•**8Clˉ exhibited a stronger photocurrent response compared to **S2•**4Clˉ or **S3•**4Clˉ upon irradiation (Fig. 3e), suggesting **1•**8Clˉ exhibited better charge separation efficiency. Correspondingly, the charge transfer resistance of **1•**8Clˉ was significantly smaller than that of **S2•**4Clˉ or **S3•**4Clˉ (Fig. 3f), further demonstrating the superior charge transfer capabilities of **1•**8Clˉ. The above results indicate that the cage structure of **1•**8Clˉ could more effectively promote the generation of ROS.

### Photocatalytic oxidation

**Figure 4.**
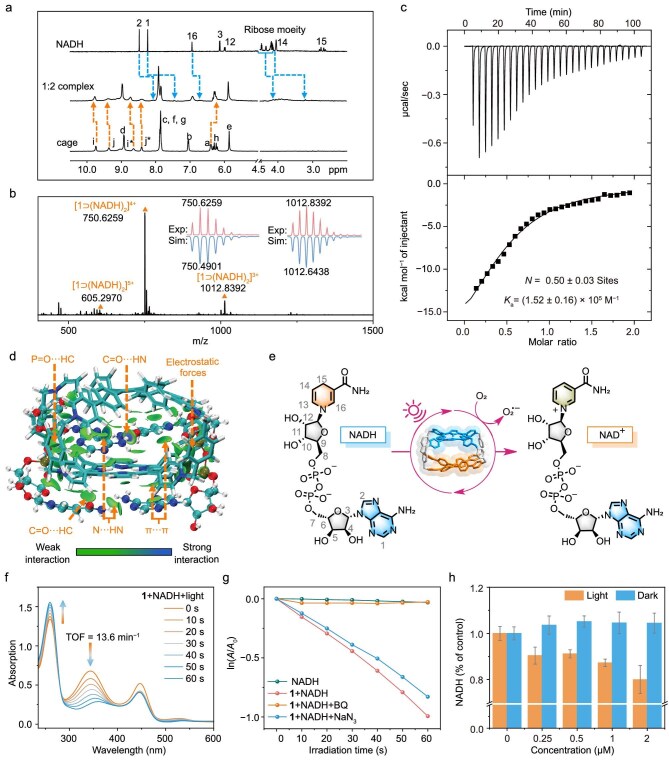
(a) Partial ^1^H NMR spectra (400 MHz, 298 K, D_2_O) recorded for **1•**8Clˉ (0.40 mM) with NADH (0.80 mM). (b) ESI-MS of **1**⊃(NADH)_2_ (Abbreviations: Exp, experiment; Sim, simulation). (c) ITC of **1•**8Clˉ (0.18 mM) titrated with NADH (0.02 mM) at 298 K in water. (d) Noncovalent interaction analysis for **1**⊃(NADH)_2_. (e) Schematic illustration of photocatalytic oxidation of NADH. (f) The absorption spectra of NADH (100 μM) in the presence of **1•**8Clˉ (10 mol%) over time upon irradiation (white LED lamp, 20 mW cm^−2^) in water. (g) Plots of ln(*A*/*A*_0_) of NADH (100 μM) at 339 nm with different catalytic agents (10 mol%) for different time intervals. (h) Quantitative analysis of intracellular NADH level in MHCC-97H cells subjected to a concentration range of **1•**8Clˉ under blue-light irradiation (300 mW cm^−2^, 20 s). Data are presented as mean ± SD derived from *n* = 5 independent experimental replicates.

NADH serves as a key reducing coenzyme in cellular metabolism, facilitating ET in numerous redox reactions. Disruption of the NADH/NAD^+^ redox equilibrium, induced through photoredox catalysis, may result in metabolic dysregulation, thereby triggering apoptosis in cancer cells. This mechanistic perturbation offers a promising strategy for targeted anticancer therapy [46,47]. The face-to-face configuration of TPE and porphyrin chromophores within the hetero-faced molecular cage enhances the photosensitive activity of the porphyrin unit. Furthermore, the molecular cage possesses a hydrophobic cavity, suggesting potential molecular recognition capabilities. We hypothesize that this molecular cage can recognize NADH in aqueous solution and, based on its photosensitive properties, catalyze the photochemical oxidation of NADH. The ^1^H NMR experiments of **1•**8Clˉ titrated with NADH in D_2_O showed that all proton resonances of NADH exhibited varying degrees of upfield shifts due to the shielding effect of the cavity, indicating that NADH may all be encapsulated within the cavity of **1•**8Clˉ (Fig. 4a and Fig. S28). For **1**, the proton resonances of TPE unit (H_a_–H_b_) and porphyrin unit (H_k_ and H_m_) exhibited upfield shifts, while the bridging CH_2_ groups (H_e_ and H_h_), pyridiniums (H_c_–H_d_ and H_i_–H_j_), and *p-*xylylene moieties (H_f_–H_g_) showed downfield shifts, caused by π-electron shielding and deshielding effects of the aromatic adenine and nicotinamide units, respectively. ESI-MS and isothermal titration calorimetry (ITC) experiments indicated the 1:2 stoichiometry with an apparent binding constant (*K*_a_) of (1.52 ± 0.16) × 10^5^ M^−1^ (Fig. 4b and c). The DFT-optimized structure of **1**⊃(NADH)_2_ elucidates the binding mode between **1** and NADH. The energy-minimized structure of **1**⊃(NADH)_2_ shows that a C=O···NH (∼1.7 Å) hydrogen bond and an N···HN (∼2.0 Å) hydrogen bond are formed between the adenine moiety of one NADH and the nicotinamide moiety of another within the cavity of **1**. The opposing nicotinamide and adenine units form a pair of N···HN (∼2.2–2.4 Å) hydrogen bonds situated outside the cavity, engaging in π···π (∼3.5 Å) interactions with the porphyrin core of **1**. Meanwhile, the phosphate group forms P = O···HC (∼2.2–2.9 Å) hydrogen bonds with *p-*xylylene unit of **1**. Multiple additional π···π and CH···π (∼2.3 Å) interactions between **1** and NADH further stabilize the host–guest complex. Independent gradient model based on Hirshfeld partition (IGMH) analysis revealed that the binding behavior between **1** and NADH is sustained by multiple noncovalent interactions, including π···π and CH···π interactions, hydrogen bonds, and electrostatic forces (Fig. 4d and Fig. S29).

Given the cage’s ROS generation and the host–guest recognition, the NADH oxidation capability of **1•**8Clˉ was further investigated in water. In this oxidative conversion reaction, the reduced nicotinamide portion of NADH loses electrons to form an electron-deficient pyridinium cation (Fig. 4e). The photocatalytic efficiency of **1•**8Clˉ toward NADH in an aqueous solution was first evaluated using UV-vis spectroscopy. The absorption peak of NADH at 339 nm decreased significantly upon irradiation in the presence of **1•**8Clˉ, indicating that **1•**8Clˉ efficiently catalyzes the oxidation of NADH to NAD^+^, with a high turnover frequency of ∼13.6 min^−1^ (Fig. 4f). ^1^H NMR spectroscopy confirmed the oxidation of NADH to NAD^+^ in the presence of **1•**8Clˉ, with characteristic NAD^+^ peaks observed under light irradiation (Fig. S30). The oxidation mechanism of NADH was compared using ROS scavengers for further analysis. The radical scavenger 1,4-benzoquinone nearly completely inhibited NADH oxidation, while the singlet oxygen scavenger sodium azide (NaN_3_) had little effect, suggesting that NADH oxidation primarily occurs through O_2_^•−^ oxidative activity (Fig. 4g). NADH depletion in MHCC-97H cells was assessed using the NAD^+^/NADH assay kit with WST-8. Upon light irradiation, intracellular NADH levels decreased with increasing **1•**8Clˉ concentration, whereas no significant change was observed in the absence of irradiation (Fig. 4h). These results underscore the potential of **1•**8Clˉ as an efficient photocatalyst for NADH oxidation, potentially improving cancer treatment efficacy.

### Photodynamic therapy

**Figure 5.**
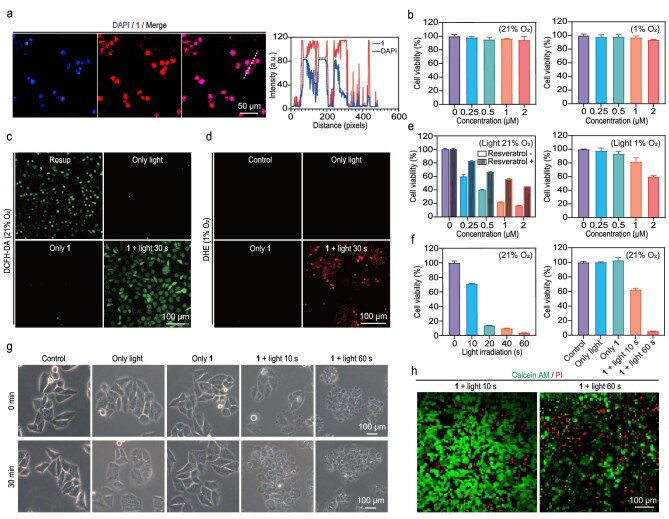
The cellular internalization and cytotoxicity of **1•**8Clˉ in MHCC-97H cells. (a) Colocalization of **1**•8Clˉ (20 μM, 24 h) and nucleus. (b) Dark toxicity detected after 30 min incubation with different concentrations of **1•**8Clˉ in normoxic and hypoxic states. Detection of ROS in (c) normoxic and (d) hypoxic states with incubation with **1•**8Clˉ at a concentration of 0.25 μM for 30 min, captured by CLSM. (e) Changes in cell viability after 10-s light exposure with or without resveratrol pretreatment, as well as the changes in cell viabilities under hypoxic condition. (f) Monitoring of cell viabilities in the presence of **1**•8Clˉ (0.25 μM) after different durations of light exposure in the normoxic state, simultaneously comparing the changes in cell survival rates under different treatment conditions. (g) Changes in cell morphology under different treatment conditions. (h) Live/Dead staining at different duration of light exposure.

To assess the anticancer efficacy of the molecular cage as a photosensitizer, we conducted further investigations into its application in PDT [48,49]. Initially, human hepatocellular carcinoma (MHCC-97H) cells were incubated with a 20 μM solution of **1•**8Clˉ. According to the results of confocal laser microscopy (CLSM) images, the **1•**8Clˉ shows good co-localization with the cell nucleus, with the Pearson coefficient as high as 0.856, indicating that this molecule not only has good permeability but also excellent nuclear targeting (Fig. 5a). Building upon the significant photodynamic activity of **1•**8Clˉ in solution, we next evaluated its capacity for intracellular ROS generation. Additionally, the dark cytotoxicity of **1•**8Clˉ was assessed under dark conditions to ensure biocompatibility. Cell viability remained near 100% even at high concentrations under both normoxic (21%) and hypoxic (1%) conditions, confirming the biological safety of **1•**8Clˉ in the absence of light exposure (Fig. 5b). Subsequently, we conducted a detailed investigation into the photodynamic effects mediated by **1•**8Clˉ. As is known, Type-I photosensitizers react directly with substrates and oxygen molecules via electron transfer, generating more active free radicals, such as superoxide anions [50]. Moreover, because of its low oxygen dependence, it can better enhance the anti-tumor effect. Therefore, to determine whether **1•**8Clˉ can induce a potent cytotoxic effect in solid tumors, it is crucial to assess its Type-I and Type-II photodynamic activities. To explore the photodynamic effects of **1•**8Clˉ, we monitored ROS generation in cells. Cells incubated with **1•**8Clˉ and DCFH-DA under dark conditions produced minimal ROS, whereas light exposure resulted in significant ROS production (Fig. 5c). Additionally, O_2_^•−^ generation was evaluated under 1% oxygen conditions. Cells incubated with **1•**8Clˉ and exposed to light exhibited intense red fluorescence, indicating significant ROS production (Fig. 5d). This fluorescence was observed only in the presence of light, while minimal to no fluorescence was detected in the dark. In contrast, cells incubated solely in the medium showed negligible fluorescence, and a similar lack of fluorescence was noted in cells exposed to light without prior incubation with **1•**8Clˉ.

Cell viability was assessed using Cell Counting Kit-8 (CCK-8) assays to further confirm the phototoxicity of **1•**8Clˉ under both normoxic and hypoxic conditions. The CCK-8 assays demonstrated that **1•**8Clˉ exhibited both Type-I and Type-II photodynamic effects, as evidenced by a time- and concentration-dependent reduction in cell viability. Additionally, the phototoxic index (PI > 5.65) indicated its strong potential for cancer therapy. At the same time, it was found that cell toxicity was partially reversed by using resveratrol to scavenge ROS without affecting NADH oxidation, which indirectly indicates that the NADH oxidation induced by the molecule also plays an important role in cell killing (Fig. 5e). Morphological alterations observed under different treatment conditions revealed that brief light exposure led to minimal changes, whereas extended exposure resulted in significant blistering and cell damage (Fig. 5f and g). Live/Dead and TUNEL staining assays further confirmed that light exposure induced apoptosis and subsequent cell death (Fig. 5h and Fig. S31). Overall, **1•**8Clˉ exhibited substantial efficacy in eradicating tumor cells under both normoxic and hypoxic conditions, underscoring its promising potential for clinical application in cancer therapy.

**Figure 6.**
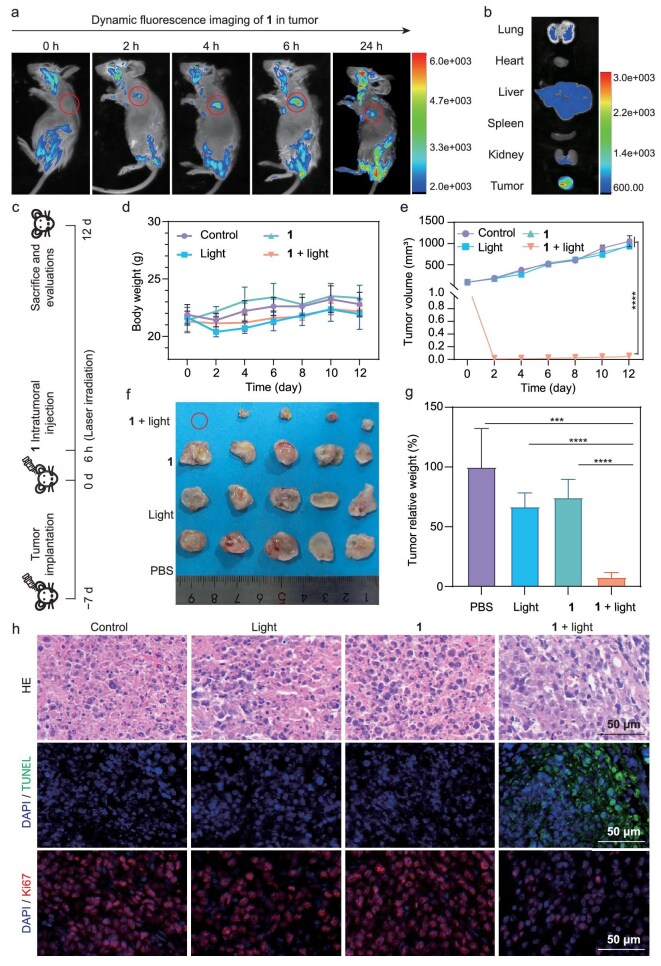
*In vivo* dynamic imaging and tumor-killing effects. (a) Dynamic distribution process after intratumoral **1•**8Clˉ (10 μg 100 μL^−1^) injection. (b) *Ex vivo* imaging of various vital organs 24 h after **1•**8Clˉ injection. (c) Flow chart of PDT treatment in tumor-bearing mice with H22 cells. (d) The body weight change curve of mice in each group during the monitoring time (*n* = 5). (e) Plots of tumor volumes in tumor-bearing mice after different treatments (*n* = 5). (f) *Ex vivo* image of mouse tumors after different treatments (*n* = 5). (g) Tumor weight after treatment in each group (*n* = 5). (h) HE staining, TUNEL staining, and Ki67 immunofluorescence of tumors in each group after treatment. ***, *P* < 0.001, and ****, *P* < 0.0001.

Given the promising biosafety and low phototoxicity of **1•**8Clˉ observed *in vitro*, its biosafety and phototoxicity *in vivo* were further evaluated. Tumor-bearing mice were established using H22 cells, and 10 μg 100 μL^−1^ of the **1•**8Clˉ solution was injected directly into the tumors. Fluorescence monitoring over time showed a gradual increase in intratumoral fluorescence intensity, reaching its peak at 6 h post-injection, which informed the optimal timing for administering PDT (Fig. 6a). After 24 h of fluorescence monitoring, key organs (heart, liver, spleen, lung, kidney, and tumor) were harvested, and fluorescence intensity measurements confirmed that the tumor exhibited significantly higher fluorescence intensity compared to other organs, indicating efficient retention of **1•**8Clˉ within the tumor (Fig. 6b). Tumor slices taken 24 h after injection into the tumor showed that its fluorescence was evenly distributed in the cytoplasm, with partial localization in the nucleus, consistent with previous cell-based observations. In addition to assessing the distribution of **1•**8Clˉ in tumors, its biosafety was also evaluated. The results demonstrated that **1•**8Clˉ exhibited favorable hemocompatibility and did not adversely affect liver or kidney function, nor did it induce significant damage to other vital organs (Figs S32–S34).

With confirmed biosafety, the next step was to evaluate the tumor-killing efficacy of **1•**8Clˉ *in vivo*. The detailed treatment protocol is as follows (Fig. 6c): the treatment protocol was established based on dynamic imaging results, which showed peak fluorescence at 6 h post-injection. A 450 nm blue laser at 3 mW cm^−2^ was used to irradiate the tumors for 5 min, 6 h following the injection of **1•**8Clˉ as part of the PDT treatment group. Over 12 days of continuous monitoring, no significant weight loss was observed in the mice across all experimental groups, indicating minimal toxicity associated with **1•**8Clˉ (Fig. 6d). Tumor volume measurements revealed that while the control groups experienced significant tumor growth, the PDT-treated group exhibited substantial reductions in tumor volume, with slower tumor progression observed over time (Fig. 6e). On day 12, the mice were euthanized, and the tumors were excised and weighed (Fig. 6f, g and Fig. S35). The excised tumor tissues were then subjected to HE staining, TUNEL staining, and Ki67 immunofluorescence staining (Fig. 6h and Fig. S36). HE staining revealed more extensive cellular damage in the PDT-treated group compared to the control groups. TUNEL staining showed an increased number of TUNEL-positive areas in the PDT-treated tumors, indicating enhanced apoptosis. Ki67 staining further confirmed that PDT effectively inhibited tumor cell proliferation. These results collectively demonstrate that **1•**8Clˉ exhibits strong biocompatibility and significant anti-tumor effects both *in vivo* and *in vitro*.

## CONCLUSION

In this study, we report the design and synthesis of a TPE-porphyrin hetero-faced molecular cage (1•8Clˉ) that addresses significant challenges in PDT applications. Traditional porphyrin-based molecular systems exhibit ACQ effects that limit their efficiency, especially in phototherapeutic contexts. By integrating TPE, a compound with AIE properties, into the porphyrin structure, we effectively convert the ACQ behavior of porphyrin into beneficial AIE characteristics. The molecular cage design, featuring a unique face-to-face configuration of TPE and porphyrin units, facilitates efficient intramolecular EnT and ET, and reduces Δ*E*_ST_ between the singlet and triplet states, finally leading to the long-lived triplet excited states. This modification enhances the generation of ROS. Furthermore, the cage structure’s hydrophobic cavity provides a platform for molecular recognition of NADH, enabling the formation of a 1:2 host–guest complex in water. This host–guest interaction promotes the oxidation of NADH to NAD^+^, catalyzed by O_2_^•−^, disrupting the biochemical equilibrium within cancer cells, including the impairment of energy metabolism and redox homeostasis that are critical for tumor cell proliferation, invasion, and metastasis, and thus contributing to the PDT’s efficacy. Combined ROS generation and the disruption of the intracellular redox balance of NADH, the cage demonstrates effective PDT in hypoxic tumor environments, a common challenge in cancer therapy that often renders conventional treatments ineffective. Such performance highlights its potential to target a broad spectrum of solid tumors characterized by hypoxic microenvironments, such as liver cancer, breast cancer, and melanoma. The findings demonstrate that this TPE-porphyrin hetero-faced cage not only overcomes the ACQ limitation but also enhances optical properties, photodynamic activity, and photocatalytic efficiency. By promoting molecular recognition and oxidative catalysis, this system provides a promising approach for future photocatalytic therapeutic strategies, particularly in targeting hypoxic tumors. The successful synthesis and design of the TPE-porphyrin hetero-faced molecular cage exemplify how structural innovation can address key challenges in PDT. The covalent molecular cage system’s enhanced ROS generation, effective molecular recognition, and catalytic properties represent significant advances in the development of high-performance therapeutic agents for cancer treatment, with promising potential for clinical applications.

## Supplementary Material

nwag241_Supplemental_File

## Data Availability

All data supporting the findings of this study are available in the article and its Supplementary information.
