# Supplementary material for: Design and synthesis of a tetraphenylethene-porphyrin hetero-faced molecular cage for photodynamic therapy
Source: Natl Sci Rev. 2026 Apr 22;13(11):nwag241. doi: 10.1093/nsr/nwag241 (PMC13263896; doi:10.1093/nsr/nwag241)
Supplement: nwag241_Supplemental_File [file nwag241_supplemental_file.pdf]

Supporting Information

Design and Synthesis of a Tetraphenylethene-Porphyrin Hetero-Faced Molecular Cage for Photodynamic Therapy

Jiaxi Fan[1,†], Zhuoxia Li[2,†], Lin Cheng[1,†,*], Zhaolong Wang[3], Zhihui Guo[1], Pingxia Wang[1], Didi Chen[4], Jiao Zhu[5], Yuting Wang[6], Jun Li[1], Xianglong Duan[6,7,*], Min Li[2,*], and Liping Cao[1,*]

[1]College of Chemistry and Materials Science, Northwest University, Xi'an 710069, China;

[2]Department of Hepatobiliary Surgery, Union Hospital, Tongji Medical College, Huazhong University of Science and Technology, Wuhan 430022, China;

[3]State Key Laboratory of Chemical Reaction Dynamics, Dalian Institute of Chemical Physics, Chinese Academy of Sciences, Dalian 116023, China;

[4]Hubei Key Laboratory of Purification and Application of Plant Anti-Cancer Active Ingredients, Hubei University of Education, Wuhan 430205, China;

[5]Department of Gastroenterology, The First Affiliated Hospital of Xi'an Jiaotong University, Xi'an, 710061, China;

[6]Shaanxi Engineering Research Center of Medical Polymer Materials, Second Department of General Surgery, Shaanxi Provincial People's Hospital, Xi'an, 710068 Shaanxi, China;

[7]Shaanxi International Science and Technology Cooperation Base for Clinical Medicine; Institute of Medical Research, Northwestern Polytechnical University; Second Department of General Surgery, Third Affiliated Hospital of Xi'an Jiaotong University. Xi'an, 710068 Shaanxi, China

*Corresponding authors. E-mails: chenglin@nwu.edu.cn; duanxianglong@nwpu.edu.cn; liminmed@hust.edu.cn; chcaoliping@nwu.edu.cn

†These authors contributed equally

Table of Contents	Pages
General experimental details	S3
Synthetic procedures and characterization data	S9
X-ray Structure determination	S17
Photophysical properties	S20
ROS generation	S25
The host-guest experiments and oxidation of NADH	S26
Experimental data *in vitro* and *in vivo*	S29

General Experimental Details

Starting materials were purchased from commercial suppliers were used without further purification. S**1**•$4Cl^-$, S**2**•$4Cl^-$**,** and S**3**•$4Cl^-$ were synthesized according to previous literatures [1–3]. Melting points were recorded by using a WRS-1A apparatus in open capillary tubes. IR spectra were measured on a TENSOR27 spectrometer. NMR spectra were recorded on a spectrometer operating at 400 MHz spectra on a Bruker ascend spectrometer. Electron Spray Ionization (ESI) mass spectra were acquired with Bruker micrOTOF-Q II electrospray instrument. UV/vis spectra were done on Agilent Cary-100 spectrometer. Fluorescence spectra were performed by using a Horiba Fluorolog-3 spectrometer. X-ray diffraction data collection was recorded by Bruker D8 Venture photon II diffractometer. The lifetime of triplet states was determined on Edinburgh LP 980 spectrometer. The time-resolved fluorescence spectra were recorded on DeltaFlex-01, excitation pulse excitation light source using Deltadiode 400 nm laser, PPD850 picosecond lifetime detector of HORIBA. Electron paramagnetic resonance (EPR) tests were performed with Bruker E500.

Theoretical Calculation.

All calculations in this study were performed using the Gaussian 16 A.03 program. First, in an aqueous environment, the ground-state geometries of all compounds were optimized employing the SMD solvation model within the self-consistent reaction field (SCRF) framework, using the B3LYP-D3/6-31G* method (with the D3 dispersion correction enabled via the "em=gd3" keyword) [4–10]. Subsequently, based on the optimized structures, the ground-state energies of the compounds were calculated at the B3LYP-D3/6-311G* level. Excited-state properties were then evaluated using time-dependent density functional theory (TD-DFT) at the CAM-B3LYP/6-31G* level of theory, which incorporated the DKH2 relativistic Hamiltonian. Spin-orbit coupling matrix elements were computed using the PySOC program [11]. The noncovalent interactions in the host–guest complex were analyzed using the independent gradient model based on Hirshfeld partition (IGMH) [12]. All isosurface maps were presented by the VMD 1.9.3 [13] program based on cube files generated by Multiwfn [14].

Biological experiments.

Materials. The human hepatocellular carcinoma cell line MHCC-97H and the mouse hepatocellular carcinoma cell line H22 were bought from the Cell Bank of the Chinese Academy of Sciences (Shanghai, China). Dulbecco's modified Eagle's medium

(DMEM) was bought from Gibco (Grand Island, USA). 2',7'-Dichlorofluorescin diacetate (DCFH-DA), penicillin–streptomycin, Cell Counting Kit-8 (CCK8), NAD^+/NADH Assay Kit with MTT and fetal bovine serum (FBS) were bought from Hycezmbio (Wuhan, China). Actin-Tracker Green (F-actin), Calcein-AM/PI dual staining kit, TUNEL apoptosis assay kit was purchased from Beyotime (Jiangsu, China). 4% paraformaldehyde and DMSO were purchased from Biosharp (Hefei, China). DAPI was purchased from Invitrogen (Carlsbad, CA, USA). The cells were imaged by confocal laser scanning microscopy (CLSM) and fluorescence microscopy. In vivo fluorescence images were recorded with a Bruker In-Vivo FX PRO.

Cell Culture. MHCC-97H cells were cultured in high-glucose DMEM medium with 10% FBS and 1% penicillin–streptomycin. And the cells were cultured at 37°C in a humidified incubator with 5% CO_2.

Cellular Imaging of 1•8Cl¯ in vitro. MHCC-97H cells in the logarithmic growth phase were uniformly inoculated in a 35 mm confocal dishes overnight. The cells were incubated with **1**•8Cl¯ (20 μM) for 24 h and washed three times with 1× PBS, and then after fixing with 4% Paraformaldehyde Fix Solution, the cells were then stained with 5 mg·mL-1 DAPI for 10 min to label the nucleus. Acquisition of images of cells were taken by confocal laser scanning microscopy (CLSM, Nikon Corporation, Japan). For **1**•8Cl¯, λ_{ex} = 405 nm, and the bandpass filter λ = 662–737 nm. For DAPI, λ_{ex} = 405 nm, and the bandpass filter λ = 425–475 nm. And imaging analysis was performed directly using NIS-Eliments software (Nikon, Japan).

Ability to Oxidize NADH. MHCC-97H cells (5×10^7 per well) were uniformly inoculated in 100 mm cell culture plates overnight, and then incubated with different concentrations (0, 0.25, 0.5, 1, 2 μM) of **1**•8Cl¯ for 30 min at 37°C. After washed with 1× PBS three times, continue culturing the cells with fresh medium and followed by blue light irradiation (300 mW cm^{-2}) for 20 s. According to the supplier's instructions, NAD^+ and NADH were extracted from the samples by acidic and alkaline extracts, respectively. NADH was reduced by oxidized thiazolyl blue (MTT) through the hydrogen transfer of PMS to produce metazoan, and NAD^+ could be reduced to NADH by ethanol dehydrogenase, and the content of NADH could be quantified through the change of absorbance value by further using the MTT reduction method. The absorbance at 450 nm was measured using a Multimode Plate Reader (PerkinElmer Pte. Ltd., Singapore).

Intracellular ROS Detection. Intracellular ROS generation ability in normoxia

(21%) and hypoxia (1%) was detected using the fluorescent probe DCFH-DA and DHE as the ROS detection kits, respectively. In brief, DCFH-DA was used as a reactive substrate for ROS in the normoxic environment while DHE was used as a reactive substrate in the hypoxic environment. The MHCC-97H cells (1×10^5 per well) were seeded in 35 mm cell culture plates overnight, cells in the normoxic and hypoxic groups were respectively loaded with 10 μM DCFH-DA and 10 μM DHE for 30 min in the dark , and then washed with PBS, then incubated with 2 μM CAGE for 30 min, and then replaced with fresh medium after washing with 1× PBS, once these steps were complete, the cells were irradiated with blue light (300 mW cm^{-2}) for 30 s, and then the fluorescence from cells was immediately captured under a fluorescence microscope (IX71, Olympus, Tokyo, Japan). The cells incubated with 50 mg mL^{-1} of a Rosup solution for 2 h were treated as positive control, the cells in only light group were treated with light irradiation for 30 s while only **1**•8Cl¯ group were incubate with **1**•8Cl¯-containing incubation solution for 30 min. For DCFH-DA, λ_{ex} = 488 nm, and the bandpass filter λ = 500–550 nm. For DHE, λ_{ex} = 561 nm, and the bandpass filter λ = 552–617 nm.

Cytotoxicity Studies in vitro. To evaluate the cytotoxicity in dark and photodynamic efficacy of CAGE against MHCC-97H cells, the cell viabilities were measured by CCK-8 assays. Briefly, the MHCC-97H cells were seeded in 96-well plates at a density of 1×10^4 cells per well, and incubated with different concentrations (0, 0.25, 0.5, 1, 2 μM) of CAGE for 30 min in normoxia (21%). After washing with 1× PBS three times, the cells were replaced with the complete medium to continually incubate for another 24 h. Each well was incubated with 100 μL of fresh medium containing 10 μL of CCK8 solution for 30 min in dark, and then the absorbance at 450 nm was measured using a Multimode Plate Reader (PerkinElmer Pte. Ltd., Singapore). The procedures of dark toxicity in hypoxia treated as described above, and the cells was cultured in 37°C at the 1% oxygen concentration for 3 h.

To assess the cytotoxicity induced by **1**•8Cl¯-mediated PDT under different concentrations in normoxia or hypoxia, the cells were incubated with different concentrations (0, 0.25, 0.5, 1, 2 μM) for 30 min in dark and then exposed to blue light irradiation (300 mW cm^{-2}) for 10 s. The cells were immediately covered with medium containing CCK8 solution for 30 min and measured the absorbance at 450 nm. The cytotoxicity in normoxia was operate under 21% O_2 throughout the entire process while the cytotoxicity in hypoxia was operated under 1% O_2.

To indirectly demonstrate that **1**•8Cl¯-mediated photodynamic therapy can oxidize NADH in tumor cells, thereby affecting their survival rate, resveratrol was used to eliminate oxygen free radicals without interfering with NADH oxidation. The cells were first pre-treated with 10 μM resveratrol for 6 hours. Subsequently, cells were incubated with varying concentrations (0, 0.25, 0.5, 1, and 2 μM) of **1**•8Cl¯ under light-shielded conditions for 30 min. After washing off residual drug, the cells were irradiated with 300 mW cm^{-2} blue light for 10 s. Immediately after irradiation, the cells were incubated with CCK8 working solution for 30 min. Finally, the absorbance at $OD_{450\ nm}$ was measured, and a cell survival rate plot was generated.

To study the cytotoxicity under different light irradiation power, the cells were incubated with 0.25 μM **1**•8Cl¯ for 30 min, and then exposed to blue light (300 mW·cm_{-2}) for different light durations. Similarly, the cell viability was measured according to the procedure above.

Calcein-AM/PI Dual Fluorescence Staining. To differentiate live and dead cells, Calcein-AM was usually used as a dye for live cells while PI was for dead cells. First, the cells were incubated with 2.5 μM **1**•8Cl¯ and washed with 1× PBS twice before light irradiation. The cells were incubated with complete medium as control group, the cells irradiated for 30 s as only light group, the cells only incubated with 2.5 μM **1**•8Cl¯ solution as only **1**•8Cl¯ group, the cells incubated with 2.5 μM **1**•8Cl¯ solution and exposed to blue light irradiation for 10 s as **1**•8Cl¯+light 10 s group, the cells incubated with 2.5 μM **1**•8Cl¯ solution and irradiated for 60 s as **1**•8Cl¯+light 60 s group. The pictures were captured by CLSM (Nikon Corporation, Japan). For Calcein-AM, λ_{ex} = 488 nm, and the bandpass filter λ = 500-530 nm. For PI, λ_{ex} = 561 nm, and the bandpass filter λ = 552-617 nm. And the ratio of live to dead cells was measured by ImageJ (National Institutes of Health Free Software, USA).

TUNEL Assay in vitro. MHCC-97H cells were seeded in 35 mm culture dishes at a density of 1×10^5 per well overnight and were incubated with 2.5 μM **1**•8Cl¯ for 30 min, washed the cells with PBS and refreshed medium to continue culturing the cells. After irradiated for different durations, the cells were fixed with 4% Paraformaldehyde Fix Solution for 15 min and permeabilized with 0.3% Triton X-100 in PBS for 10 min. Washed with 1× PBS three times, the cells were incubated with a one-step TUNEL apoptosis reaction mixture for 40 min in the dark. After removing excess dye, labeled the nucleus with DAPI for 10 min. Observed the fluorescence under a CLSM (Nikon Corporation, Japan). For TUNEL, λ_{ex} = 488 nm, and the bandpass filter λ = 500–530

nm. For DAPI, λ_{ex} = 405 nm, and the bandpass filter λ = 425–475 nm. And the relative fluorescence intensity of control was measured by ImageJ (National Institutes of Health Free Software, USA).

Animal Model. Male BALB/c mice (4 weeks old) were purchased from Biont Biotechnology Co., Ltd. (Wuhan, China). All mice were raised under specific pathogen-free (SPF) conditions with a 12/12 h dark/light cycle. We used animal isograft model studies according to institutional guidelines. The appropriate amount of H22 cells was injected into the abdominal cavity of mice, and after the ascites grew, the ascites was extracted and the concentration of cells was adjusted to 1×10^6, and finally the cells were injected into the subcutaneous of the mice to establish a tumor-bearing model. When the tumors reached an average of 60 mm^3, the mice were randomly divided into four groups (n = 5 mice in each group), and this day was designated as day 0. The **1**•8Cl^- was intratumorally injected into the tumors, and the diameters of the tumors and body weights were measured every two days. Tumor volume was calculated using the following formula: volume = ((tumor length) × (tumor width)^2)/2). Mice were exposed to a 450 nm blue laser irradiation (3 $W\cdot cm^{-2}$) for 5 min at 6 h after **1**•8Cl^- injection. Mice were euthanized 12 days after injection. The tumors were obtained and weighed following necropsy. All animal experiments were approved by the Ethics Committee of the Union Hospital of Huazhong University of Science and Technology ([2024] IACUC Number: 4760) and were performed in accordance with the guidelines of the Department of Laboratory Animals of Tongji Medical College.

In vivo Fluorescence Imaging and Biodistribution Analysis of 1•8Cl^-. As it mentioned before, we successfully established a tumor-bearing mouse model of H22 cells. When the mean tumor volume reached approximately 60 mm^3, 100 μL of **1**•8Cl^- solution was intratumorally injected evenly into the tumor (10 μg 100 μL^{-1}). After administration, we performed real-time fluorescence imaging of mouse tumors 0, 2, 4, 6, and 24 h post-injection using an In-Vivo FX PRO (Bruker, Germany). Other mice were sacrificed 24 h after continuous monitoring, and the fluorescence intensity of various organs (liver, kidney, spleen, heart, and lung), and tumors were measured using an In-Vivo FX PRO (Bruker, Germany). And tumor sections were observed after incubation with DAPI by CLSM (Nikon Corporation, Japan).

Intratumoral Distribution of 1•8Cl^- within Tumor Tissue. After establishing the H22 cells tumor-bearing model followed by the intratumoral injection of **1**•8Cl^- for 24 h, the tumors were stripped from the mice and frozen in liquid nitrogen. Frozen

tumor tissues were sectioned into 3 μm slices and fixed with 4% paraformaldehyde for 4 h. Nucleus were stained with DAPI for 30 min. The slices were observed by CLSM (Nikon Corporation, Japan) to monitor the fluorescence intensity and tissue distribution of **1**•8Cl^{-}.

In vivo Biosafety Evaluation Analysis of 1•8Cl^{-}. H22 cells tumor-bearing mice were randomly divided into two groups, one of which was injected with 100 μL of PBS intratumorally as the control group, while another group was injected with 10 μg/100 μL **1**•8Cl^{-} as the **1**•8Cl^{-} group. After 24 h of injection, 200 μL of blood samples were taken from each mouse orbit. All blood samples were allowed to stand at room temperature for 2 h, and then the supernatant was removed and stored at −80°C. The serum levels of alanine aminotransferase (ALT), aspartate aminotransferase (AST), lactate dehydrogenase (LDH), blood urea nitrogen (BUN), creatinine (CREA), and uric acid (UA) were evaluated using a liver or renal function activity assay (Servicebio Technology, China). To further explore the biosafety of CAGE in vivo, the main organs of the mice, containing the lungs, liver, spleen, and kidneys, were extracted for H&E staining and analyses.

In vivo Anti-tumor Study and Immunohistochemical Fluorescence Staining. The H22 cells tumor-bearing mice models were performed as described above. When the tumor size reached 60 mm^3, the mice were randomly divided into 4 groups (n=5): Group 1 also named Negative Control, only intratumorally injected PBS; Group 2 called Light group: which only accept blue laser irradiation (450 nm, 3 W·cm^{-2}, 5 min) on day 0; Group 3, **1**•8Cl^{-} group: 100 μL of **1**•8Cl^{-} (containing **1**•8Cl^{-} 10 μg 100 μL^{-1}) injected intratumorally then protection of the mice in the dark; and Group 4 called **1**•8Cl^{-}+Light group: 100 μL of **1**•8Cl^{-} (50 μg 100 μL^{-1}) injected intratumorally then accepted to blue laser irradiation (450 nm, 3 W cm^{-2}, 5 min) on day 0. Twelve days after injection, the mice were sacrificed.

Tumor tissues were all collected and kept in 4% paraformaldehyde, embedded in paraffin and cut into 3 μm sections, which were deparaffinized and rehydrated subsequently. Antigen retrieval was performed at high temperatures in a pressure cooker using citrate buffer (pH 6.0). Primary antibodies against Ki67 (1:400, Proteintech) was used for immunohistochemical staining. Slices cut from the paraffin sections were stained with hematoxylin and eosin (H&E) and subjected to TUNEL before scanning with a fluorescence microscope (IX71, Olympus, Tokyo, Japan). Ki67 relative fluorescence intensity analysis results were evaluated using ImageJ (National

Institutes of Health Free Software, USA).

Statistical Analysis. Data are presented as the means ± standard error of the mean (s.e.m.) or standard deviation (SD), as indicated in the figure legends. One-way ANOVA with Tukey's multiple comparisons test was used for multiple comparisons when more than two groups were compared, and two-tailed Student's t tests were used for two-group comparisons. For all statistical analyses, values of $P < 0.05$ were considered significant. All statistical tests were performed using GraphPad Prism software v. 9.5.0 (GraphPad Software). The Student's t-test was performed to analyze the assay results. In all experiments, p value < 0.05 was considered as a statistically significant difference ('*' as presented). Among them, *** denoted $P < 0.001$, while **** signified $P < 0.0001$.

Synthetic Procedures and Characterization Data

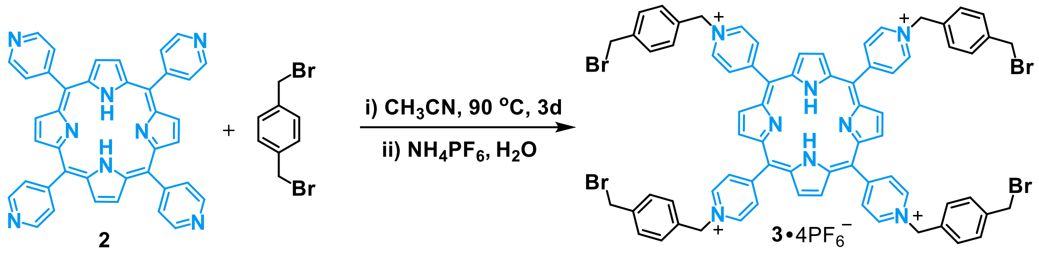

***Compound 2•4PF$_6^-$*.** 1,4-bis(bromomethyl)benzene (640 mg, 2.43 mmol) was added to dry MeCN (100 mL) in two-necked flask and the suspension was heated at 90 °C until all compound was dissolved. Compound **2** (100 mg, 0.16 mmol) was added to the MeCN solution of **S2** in batches and heated to 90°C for another 3 days. Then the mixture was cooled to room temperature, and the precipitate was collected and washed with an excess amount of acetone (3 × 40 mL) by centrifuge to give crude product with Br^- counterions as a yellow solid. Crude product with excess amount of NH_4PF_6 in H_2O (2 mL) was dissolved and stirred for 12 h. The precipitate was collected and washed with an excess amount of H_2O (3 × 20 mL) to give **3**•4PF$_6^-$ as a yellow solid (197 mg, 65%). M.p. > 300°C. IR (KBr, cm^{-1}): 3440s, 1637s, 1600m, 1498w, 1246w, 1161w, 841s, 557m. ^{1}H NMR (400 MHz, CD_3CN): 9.22 (d, J = 6.7, 8H), 9.08 (s, 8H), 8.85 (d, J = 6.7, 8H), 7.80 (d, J =8.2, 8H), 7.74 (d, J = 8.2, 8H), 6.11 (s, 8H), 4.74 (s, 8H), -3.05 (s, 2H). ^{13}C NMR (100 MHz, CD_3CN): 158.4, 143.3, 140.5, 133.3, 132.9, 130.4, 130.0, 127.8, 115.7, 64.2, 63.2, 32.8 (only 12 of the 14 resonances expected were observed due to the similar chemical environment for porphyrin ring). ESI-TOF-MS: m/z 645.5828 ([**3**•4PF$_6^-$ – 2PF$_6^-$]$^{2+}$, calcd. for $[C_{66}H_{55}Br_3N_3OP_2F_{12}]^{2+}$, 645.5737); 382.0728 ([**3**•4PF$_6^-$ – 2PF$_6^-$]$^{3+}$, calcd. for $[C_{66}H_{55}Br_3N_3OPF_6]^{3+}$, 382.0708).

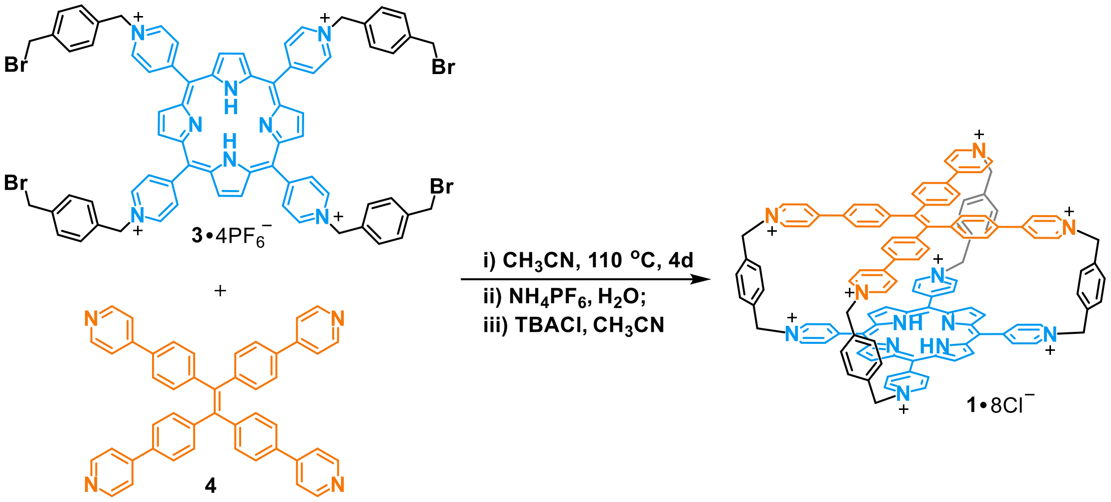

Synthetic route 1

Compound 1•8PF$_6^-$. Compound **3**•4PF$_6^-$ (100 mg, 53.2 μmol) and tetrabutylammonium iodide (TBAI, 3.3 mg, 9.1 μmol) were added to dry MeCN (200 mL) in sealed tube and the suspension was heated at 90°C. Compound **4** (41 mg, 63.9 μmol) was added to the suspension of **3**•4PF$_6^-$ in batches and heated to 110 °C for another 4 days. Then the mixture was cooled to room temperature, and the precipitate was collected and washed with an excess amount of MeCN (3 × 40 mL) by centrifuge to give crude product with Br$^-$ counterions as a yellow solid. Crude product with excess amount of NH_4PF_6 in H_2O (10 mL) was dissolved and stirred for 12h. The precipitate was collected and washed with an excess amount of H_2O (3 × 20 mL) to give crude product with PF$_6^-$ counterions as red solid. The crude product was purified by silica gel chromatography with CH_2Cl_2: MeCN (saturated NH_4PF_6) = 4:1 (v:v) as mobile phase (8 mg, 5.3%). M.p. > 300°C. IR (KBr, cm^{-1}): 3440s, 1637s, 1600m, 1498w, 1246w, 1161w, 841s, 557m. ^{1}H NMR (400 MHz, CD_3CN): 9.44(d, J = 6.0, 4H), 9.18 (d, J = 5.6, 4H), 8.98 (d, J = 7.9, 4H), 8.73 (d, J = 6.4, 8H), 8.00 (d, J = 6.4, 4H), 7.93 (d, J = 6.4, 4H), 7.80 (d, J = 6.7, 8H), 7.77 (s, 24H), 7.02 (d, J = 7.9, 8H), 6.32 (d, J = 7.2, 8H), 6.13 (d, J = 14.7, 4H),6.03 (d, J = 14.6, 4H), 5.72(s, 8H), -3.25 (s, 2H). ^{13}C NMR (100 MHz, CD_3CN): 157.4, 156.1, 144.8, 144.2, 143.3, 140.8, 139.4, 136.6, 134.7, 133.1, 132.8, 132.3, 131.4, 130.7, 130.1, 127.4, 125.7, 115.3, 63.5, 63.3 (only 20 of the 22 resonances expected were observed due to the similar chemical environment for porphyrin ring). ESI-TOF-MS: m/z 800.1947 ([**1**•8PF$_6^-$ – 3PF$_6^-$]$^{3+}$, calcd. for [$C_{118}H_{90}N_{12}P_5F_{30}$]$^{3+}$, 800.1879); 563.8997 ([**1**•8PF$_6^-$ – 4PF$_6^-$]$^{4+}$, calcd. for [$C_{118}H_{90}N_{12}P_4F_{24}$]$^{4+}$, 563.9030); 422.1343 ([**1**•8PF$_6^-$ – 5PF$_6^-$]$^{5+}$, calcd. for [$C_{118}H_{90}N_{12}P_3F_{18}$]$^{5+}$, 422.3275); 327.6199 ([**1**•8PF$_6^-$ – 6PF$_6^-$]$^{6+}$, calcd. for

$[C_{118}H_{90}N_{12}P_2F_{12}]^{6+}$, 327.6116); 260.1081 ([**1**•8PF_6^- − 7PF_6^-]$^{7+}$, calcd. for $[C_{118}H_{90}N_{12}P_1F_6]^{7+}$, 260.1007).

Compound 1•8Cl⁻. The solution of **1**•8PF_6^- (100 mg, 74 μmol) in MeCN (10 mL) was added excess amount of tetrabutylammonium chloride hydrate (206 mg, 0.74 mmol), and the mixture was stirred for another 12h. The precipitate was collected and washed with an excess amount of MeCN (3 × 10 mL) to give **1**•8Cl^- as a red solid (63.5 mg, 92%). M.p. > 300°C. IR (KBr, cm^{-1}): 3408s, 1647s, 1637s, 1599s, 1492m, 1596m, 1403w, 1296m, 1164m, 1010m, 793s, 755w. ^{1}H NMR (400 MHz, DMSO-d_6): 9.93 (d, J = 4.8, 4H), 9.47 (d, J = 3.8, 4H), 9.31 (d, J = 6.3, 8H), 9.23 (s, 4H),9.12 (s, 4H), 8.32 (d, J = 5.7, 4H), 8.13 (d, J = 6.0, 8H), 8.06 (d, J = 5.4, 4H), 7.96 (d, J = 8.1, 8H), 7.89 (d, J = 8.2, 8H), 7.30 (s, 8H), 6.35(s, 8H), 6.29(d, J = 15.1, 4H), 6.13(d, J = 14.1, 4H), 5.9 (s, 8H), -3.24 (s, 2H). ^{13}C NMR (100 MHz, CD_3OD): 157.7, 156.2, 145.5, 144.7, 143.9, 141.5, 140.2, 137.0, 134.7, 132.9, 132.0, 132.1, 131.6, 130.3, 127.6, 125.6, 116.0, 78.1, 63.6, 63.4 (only 20 of the 22 resonances expected were observed due to the similar chemical environment for porphyrin ring). ESI-TOF-MS: m/z 605.1914 ([**1**•8Cl^- − 1HCl − 3Cl^-]$^{3+}$, calcd. for $[C_{118}H_{89}N_{12}Cl_4]^{3+}$, 605.2010); 445.1640 ([**1**•8Cl^- − 1HCl − 4Cl^-]$^{4+}$ calcd. for $[C_{118}H_{89}N_{12}Cl_3]^{4+}$, 445.1599); 436.1648 ([**1**•8Cl^- − 2HCl − 4Cl^-]$^{4+}$ calcd. for $[C_{118}H_{88}N_{12}Cl_2]^{4+}$, 436.1658);349.1299 ([**1**•8Cl^- − 1HCl − 5Cl^-]$^{5+}$ calcd. for $[C_{118}H_{89}N_{12}Cl_2]^{5+}$, 349.1349); 341.7411 ([**1**•8Cl^- − 2HCl − 5Cl^-]$^{5+}$ calcd. for $[C_{118}H_{88}N_{12}Cl]^{5+}$, 341.7390);284.9486 ([**1**•8Cl^- − 1HCl − 6Cl^-]$^{6+}$ calcd. for $[C_{118}H_{89}N_{12}Cl]^{6+}$, 285.9505);278.9565 ([**1**•8Cl^- − 2HCl − 6Cl^-]$^{6+}$ calcd. for $[C_{118}H_{88}N_{12}]^{6+}$, 278.9542).239.2472 ([**1**•8Cl^- − 1HCl − 7Cl^-]$^{7+}$ calcd. for $[C_{118}H_{89}N_{12}]^{7+}$, 239.2477).

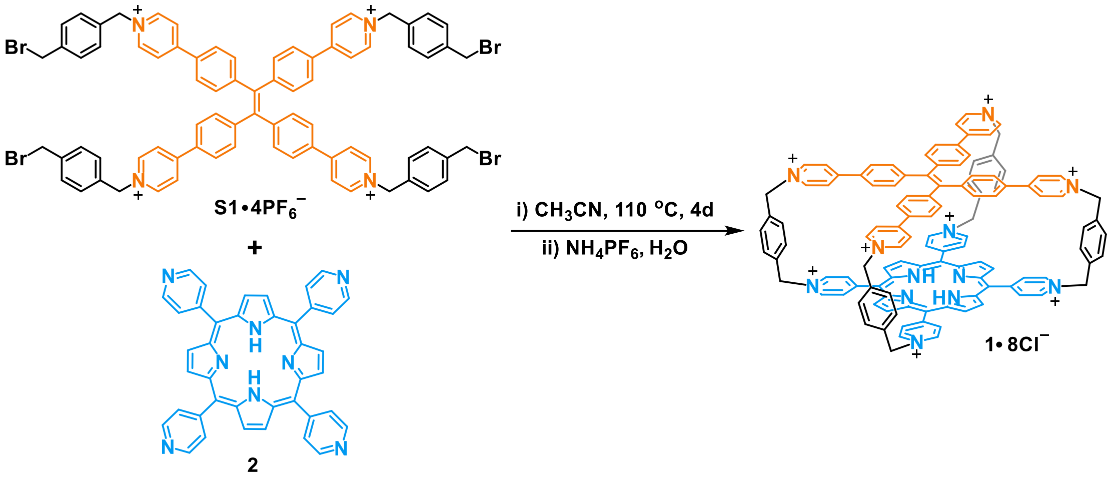

Synthetic route 2

Compound 1•8PF₆⁻. Compound **S1**•4PF_6^- (100 mg, 51.10 μmol) and TBAI (3.2

mg, 8.8 μmol) were added to dry MeCN (200 mL) in sealed tube and the suspension was heated at 90°C. Compound **2** (38 mg, 61.4 μmol) was added to the suspension of **S1**•4PF_6^- in batches and heated to 110 °C for another 4 days. Then the mixture was cooled to room temperature, and the precipitate was collected and washed with an excess amount of MeCN (3 × 40 mL) by centrifuge to give crude product with Br^- counterions as a yellow solid. Crude product with excess amount of NH_4PF_6 in H_2O (10 mL) was dissolved and stirred for 12h. The precipitate was collected and washed with an excess amount of H_2O (3 × 20 mL) to give crude product with PF_6^- counterions as red solid. The crude product was purified by silica gel chromatography with CH_2Cl_2:MeCN (saturated NH_4PF_6) = 4:1 (v:v) as mobile phase (0.48 mg, 0.32%).

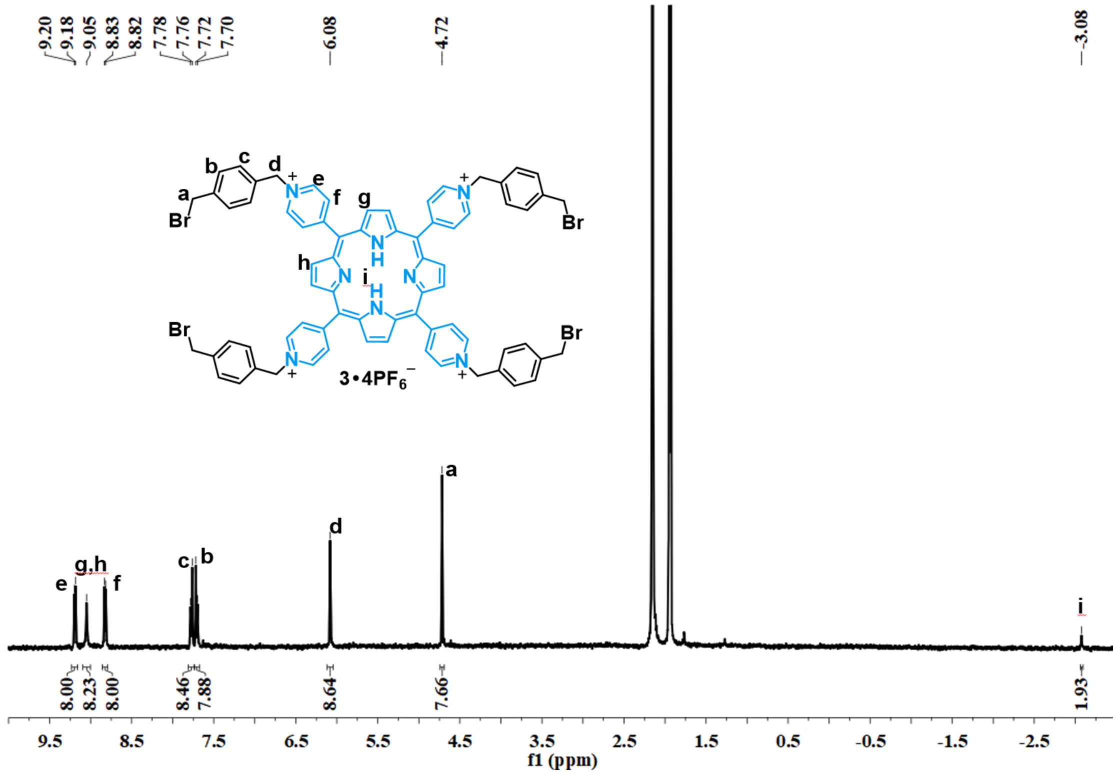

Figure S1. ^{1}H NMR spectrum recorded (400 MHz, CD_3CN, 298 K) for **3**•$4PF_6^-$.

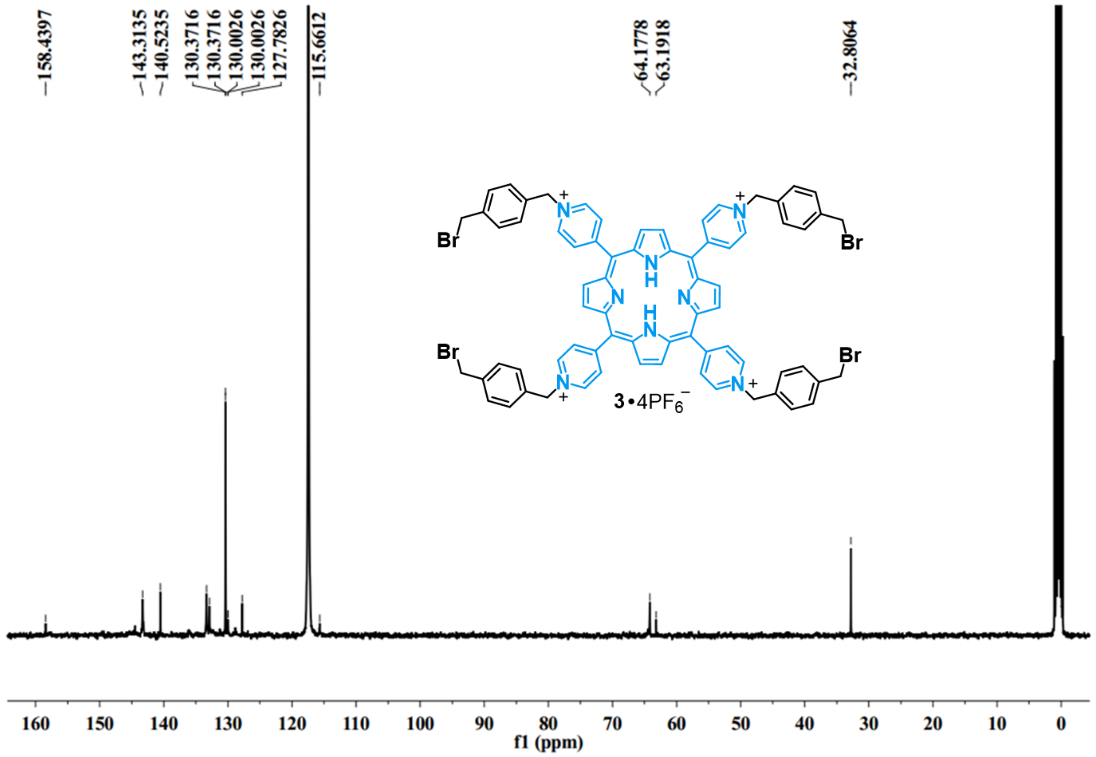

Figure S2. ^{13}C NMR spectrum recorded (100 MHz, CD_3CN, 298 K) for **3**•$4PF_6^-$.

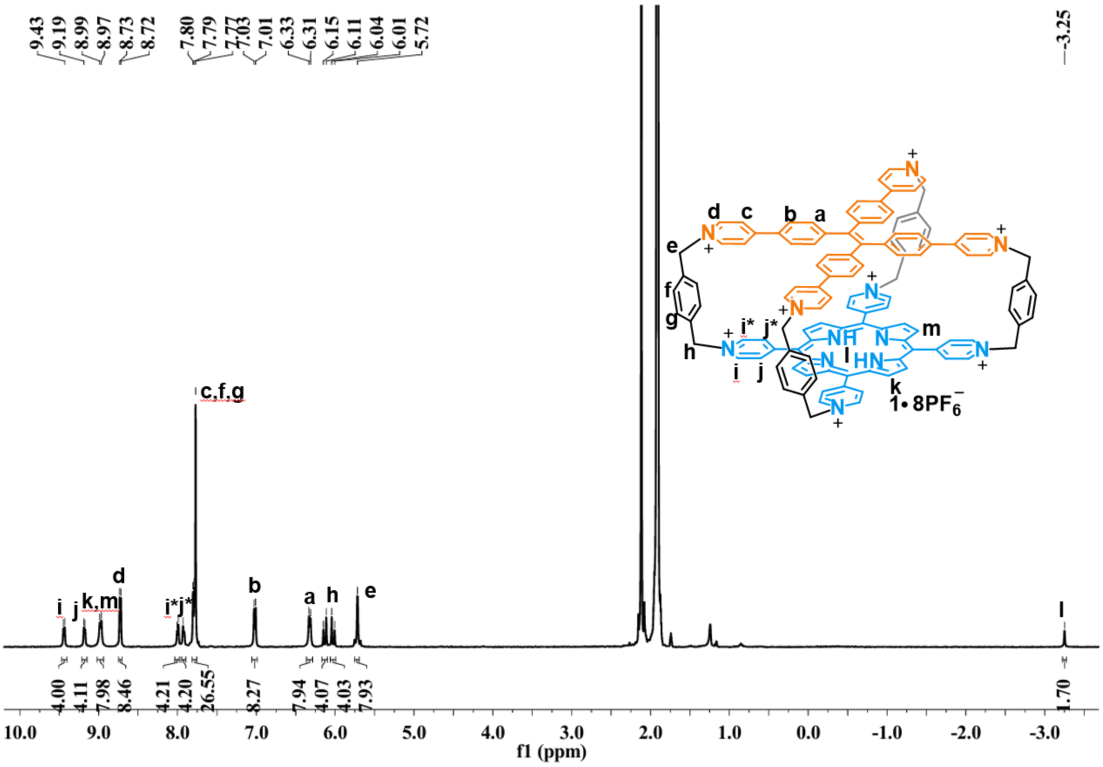

Figure S3. ^{1}H NMR spectrum recorded (400 MHz, CD_3CN, 298 K) for **1**•$8PF_6^-$.

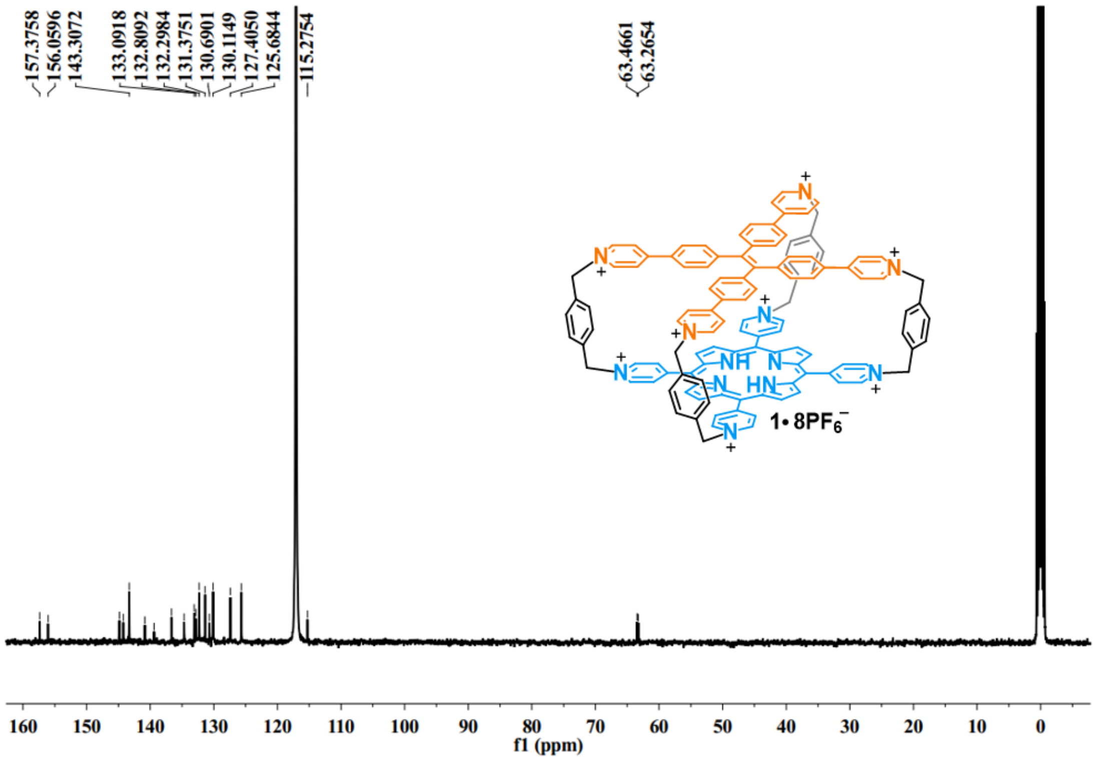

Figure S4. ^{13}C NMR spectrum recorded (100 MHz, CD_3CN, 298 K) for **1**•$8PF_6^-$.

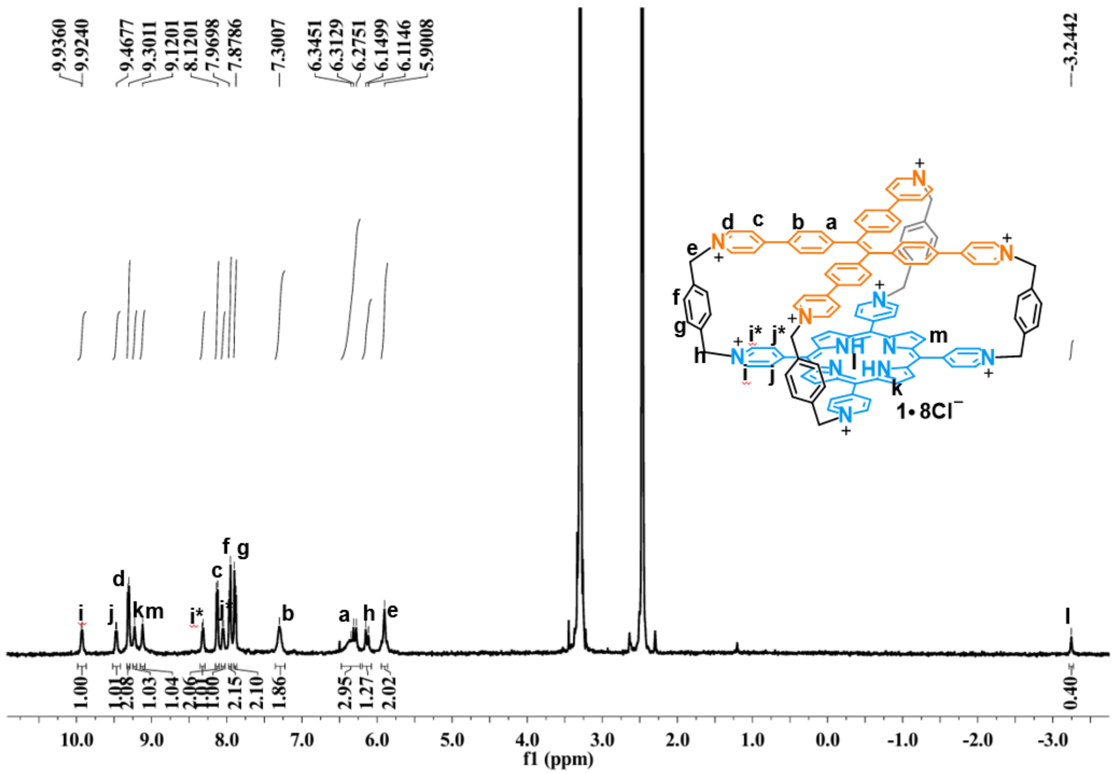

Figure S5. ^{1}H NMR spectrum recorded (400 MHz, DMSO-d_6, 298 K) for **1**•8Cl^{-}.

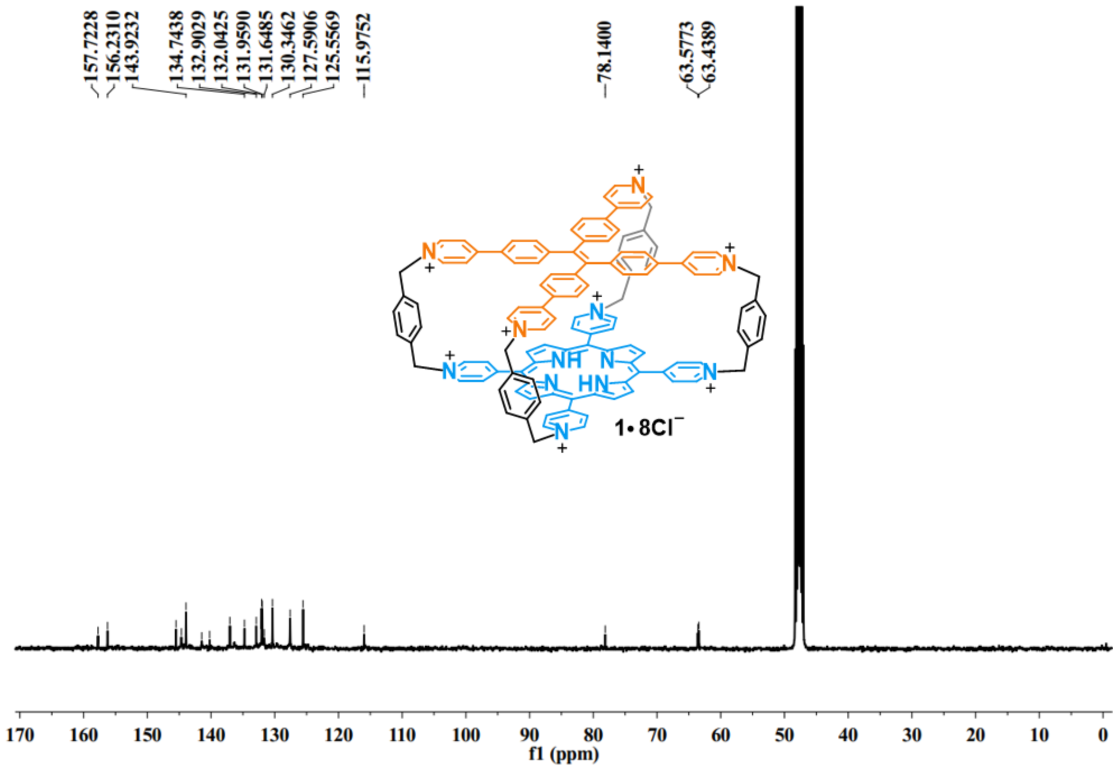

Figure S6. ^{13}C NMR spectrum recorded (100 MHz, CD_3OD, 298 K) for **1**•8Cl^{-}.

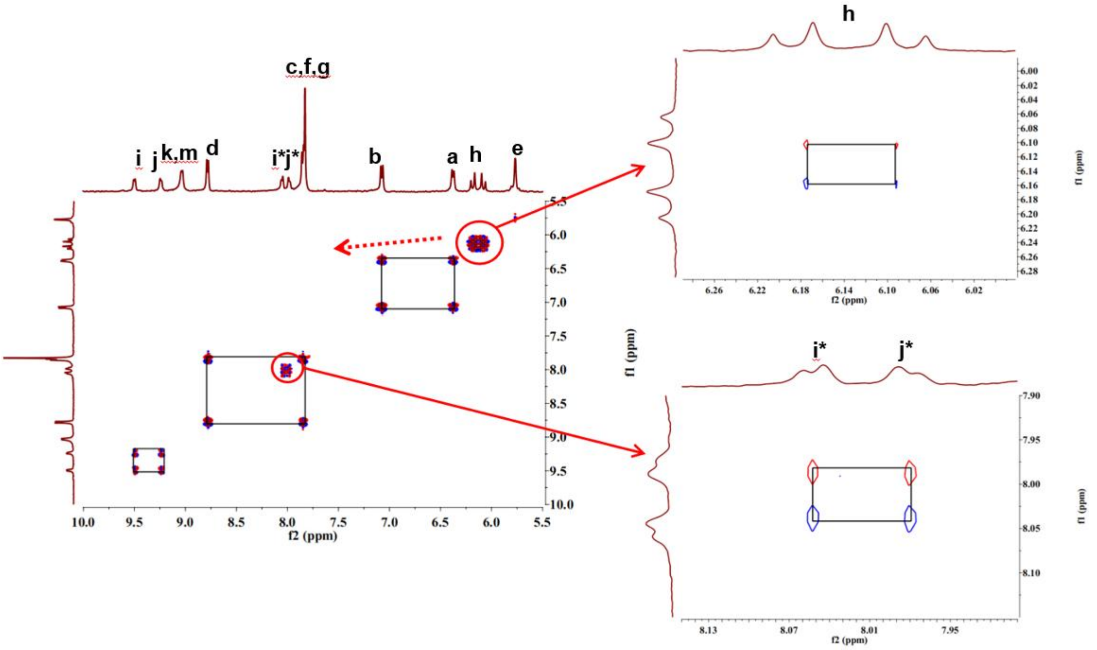

Figure S7. COSY 2D spectrum recorded (400 MHz, CD_3CN, 298 K) for **1**•8PF_6^-.

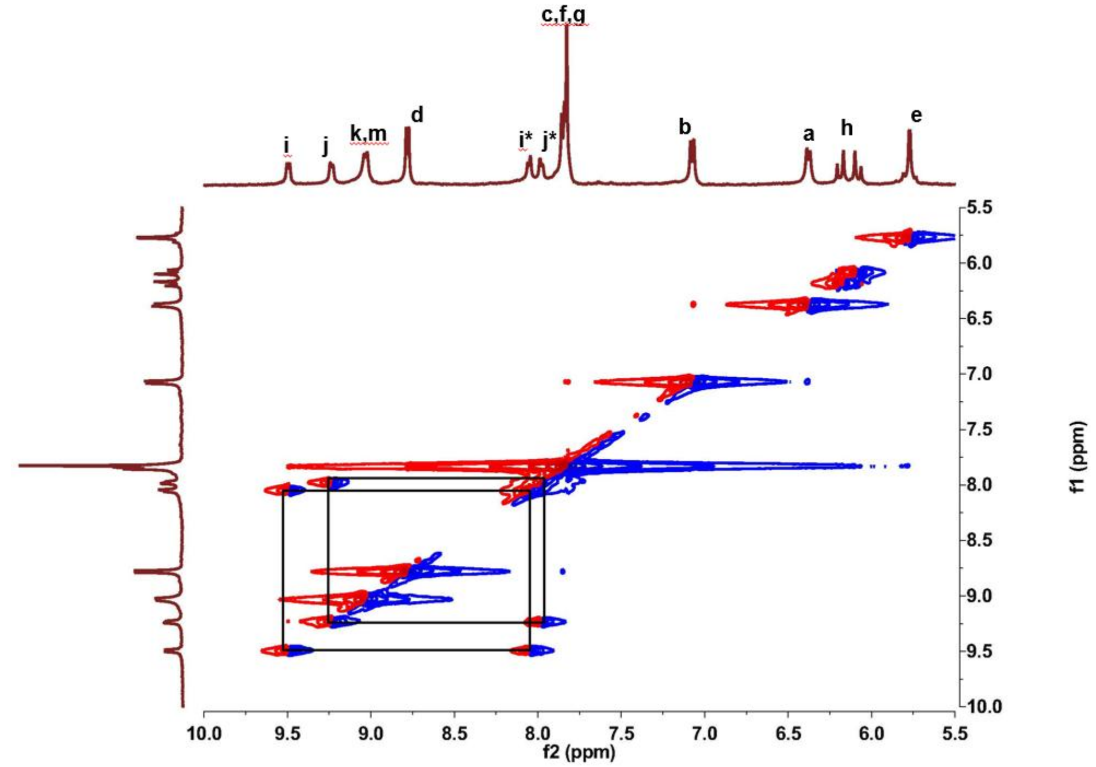

Figure S8. NOESY 2D spectrum recorded (400 MHz, CD_3CN, 298 K) for **1**•8PF_6^-.

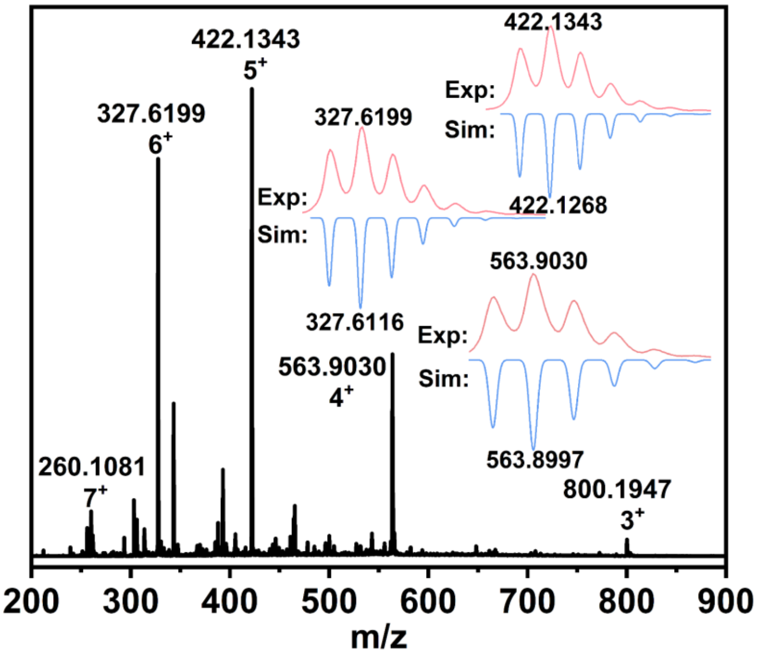

Figure S9. Experimental and calculated electrospray ionization mass spectra of **1**•8PF_6^-.

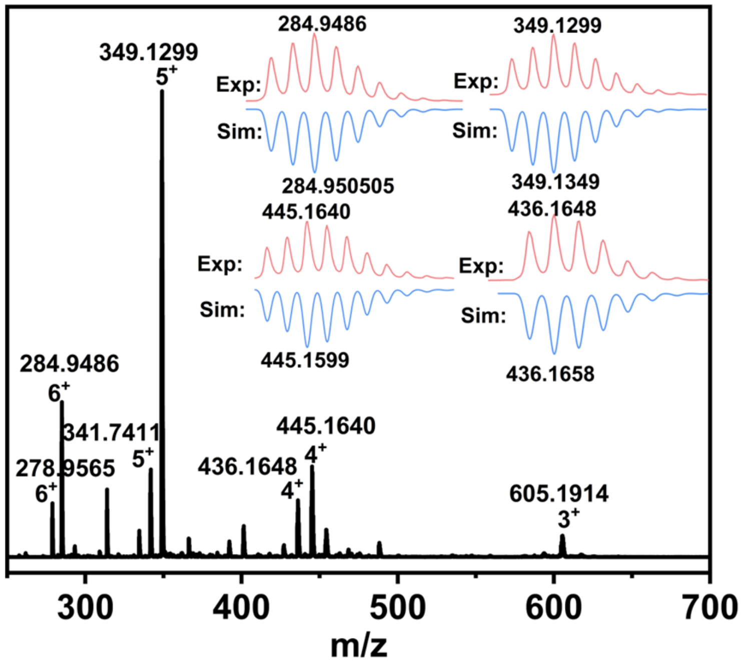

Figure S10. Experimental and calculated electrospray ionization mass spectra of **1**•8Cl^-.

X-ray Structure determination.

X-ray diffraction data collection of the compounds was recorded by Bruker D8 Venture photon II diffractometer at 173 K with graphite-monochromated Mo Kα radiation (λ = 0.71073 Å) and Bruker VENTURE system with PHOTON II CPAD detector equipped at 180 K and a Ga-target Liquid METALJET D2 PLUS X-ray Source (λ = 1.34139 Å). The structure was solved by SHELXT (version 2018/2) and refined by full-matrix least-squares procedures using the SHELXL program (version 2018/3) through the OLEX2 graphical interface.

The crystal of 1•8Cl^{-}: 1•8Cl^{-} (0.49 mg, 0.5 mmol) was dissolved in CH_3OH (0.5 mL) and the solution was passed through a 0.45 μm filter into a 10 mL tube, which was placed inside a 500 mL wild-mouth bottle containing isopropyl ether (50 mL). The bottle was capped, after slow evaporation of diethyl ether at room temperature into the CH_3OH solution for 3 days, and dark red single crystals of **1**•8Cl^{-} were obtained.

Table S1. Crystal data and structure refinement for **1**•8Cl^{-}.

Empirical formula	$C_{118}H_{90}N_{12}Cl_8$
Formula weight	1959.63
Temperature	146 K
Wavelength	1.54178 Å
Crystal system, space group	Monoclinic, $P2_1/c$
Unit cell dimensions	a=20.164（8）Å α=90°<br>b=23.608（10）Å β=103.4°<br>c=31.583（13）Å λ=90°
Volume	14623（10）Å^3
Z, Calculated density	4, 0.890 mg/m^3
Absorption coefficient	1.714 mm^{-1}
F(000)	4072
Crystal size (mm^3)	0.24×0.28×0.38
Theta range for data collection	48.95-5.80
Reflections collected/unique	26805/26757
Completeness to θ = 68.244	99.8%
Absorption correction	Multi-Scan

Max. and min. transmission	0.754 and 0.590
Refinement method	Reported T Limits
Data/restraints/parameters	26757/0/1279
Goodness-of-fit on F^2	1.064
Final R indices [I>2sigma(I)]	$R_1 = 0.1347$, $wR_2 = 0.3214$
R indices (all data)	$R_1 = 0.1840$, $wR_2 = 0.3498$
Extinction coefficient	109324/26757
Largest duff. peak and hole	1.56 and -0.78
CCDC	2477030

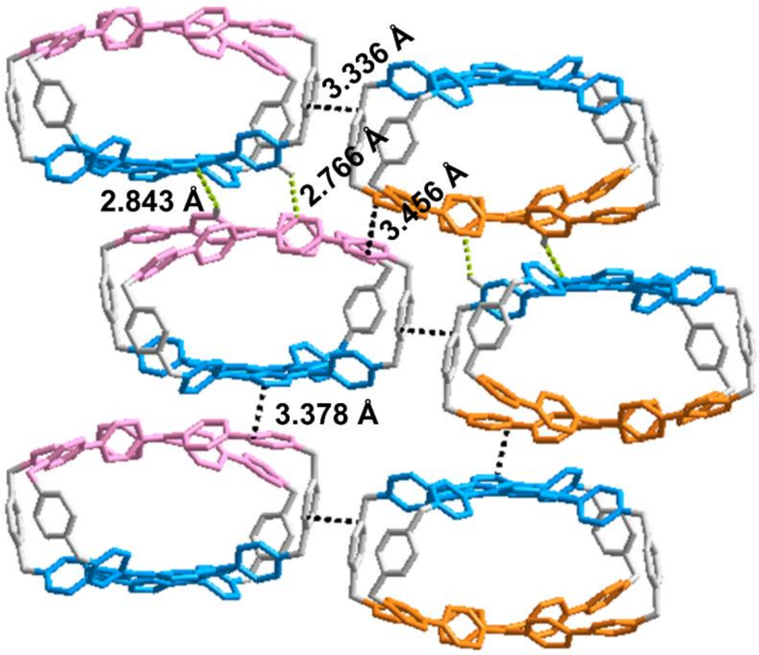

Figure S11. The π···π and C-H···π interactions between **1**•8Cl$^-$ molecules in the stacking structure.

Photophysical properties

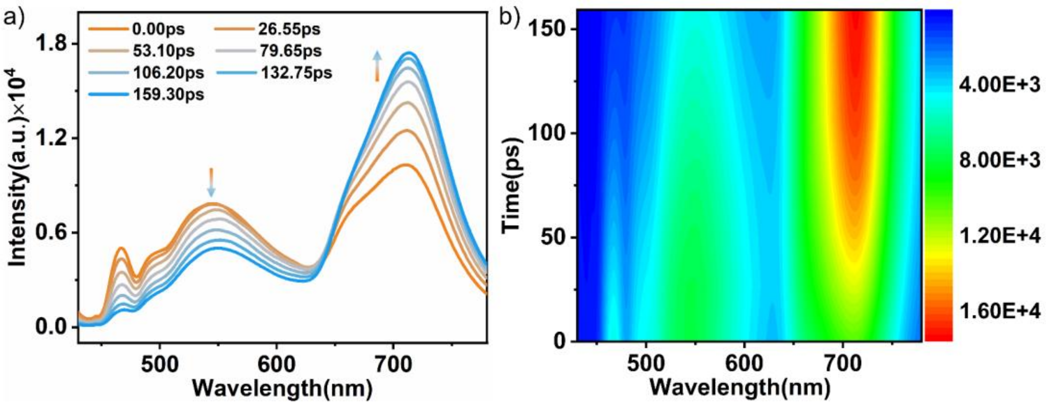

Figure S12. Time-resolved fluorescence spectra of **1**•8Cl^{-}: (a) 1D spectra and (b) 2D spectra. λ_{ex} = 400 nm. The time when the emission peak of **1**•8Cl^{-} reached the maximum value was set to 0.00 ps.

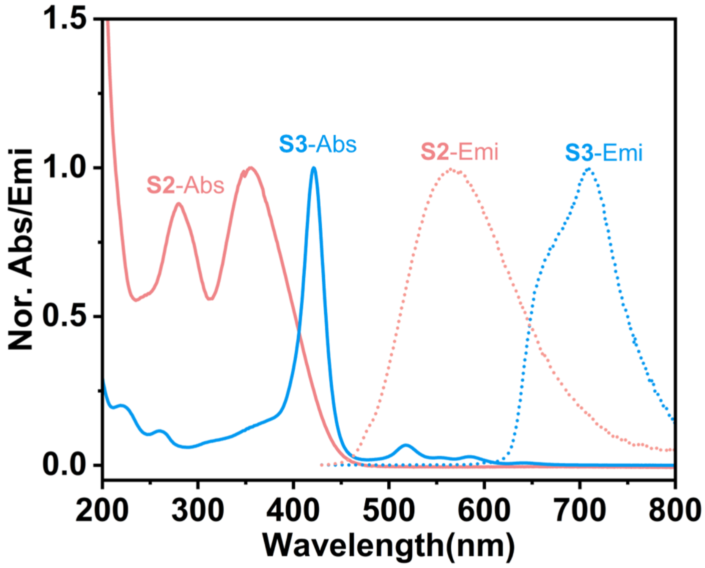

Figure S13. UV/vis absorption and Fluorescence spectra of **S2**•4Cl^{-} and **S3**•4Cl^{-} (10 μM) in H_2O. λ_{ex} = 400 nm, E_x/E_m slit = 3 nm.

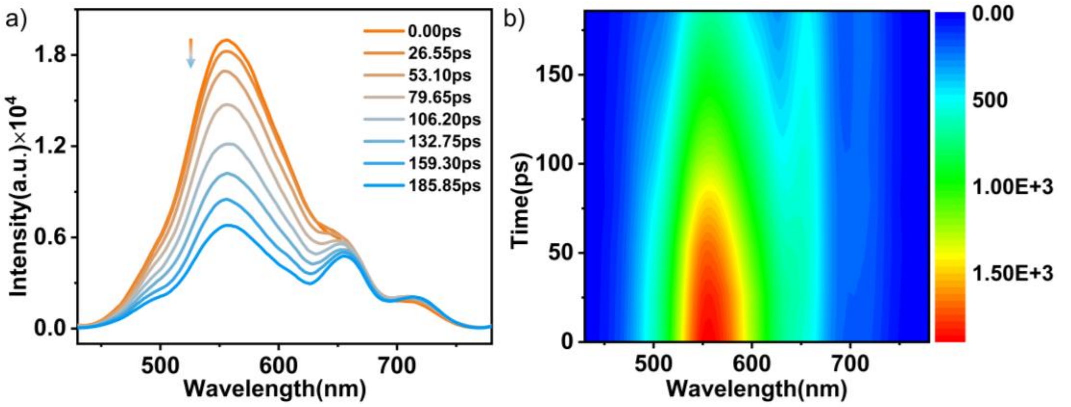

Figure S14. (a) 1D and (b) 2D time-resolved fluorescence spectra of the 1:1 mixture of **S2**•4Cl^{-} and **S3**•4Cl^{-}. λ_{ex} = 400 nm. The time when the emission peak reached the maximum value was set to 0.00 ps.

Table S2. Fluorescence decay lifetimes of **1**•8Cl^{-}, **S2**•4Cl^{-}, and **S3**•4Cl^{-} in H_2O.

Compound	τ(ns) 550 nm	τ(ns) 700 nm
**1**•8Cl^{-}	4.30	3.87
**S2**•4Cl^{-}	2.08	–
**S3**•4Cl^{-}	–	5.48

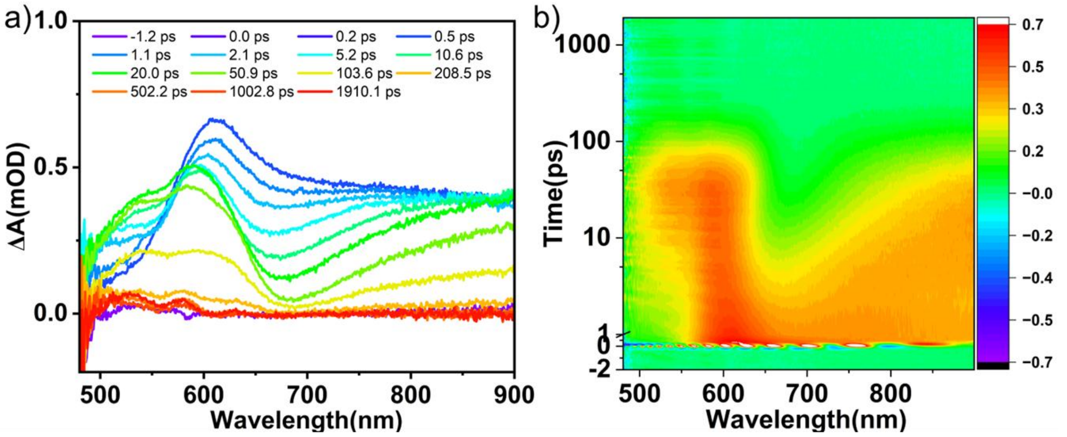

Figure S15. Femtosecond transient absorption spectra of **S2**•4Cl^{-} (10 μM) in water. λ_{ex} = 400 nm

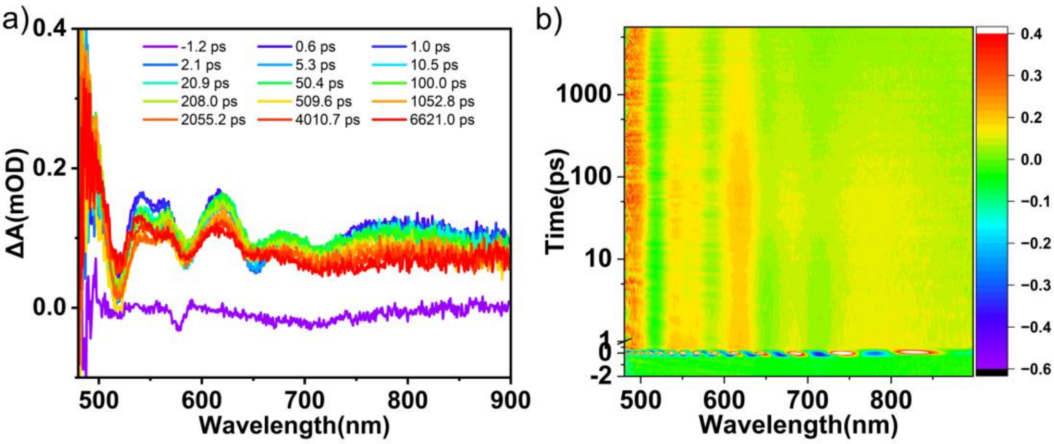

Figure S16. Femtosecond transient absorption spectra of **S3**•4Cl^{-} (10 μM) in water. λ_{ex} = 400 nm

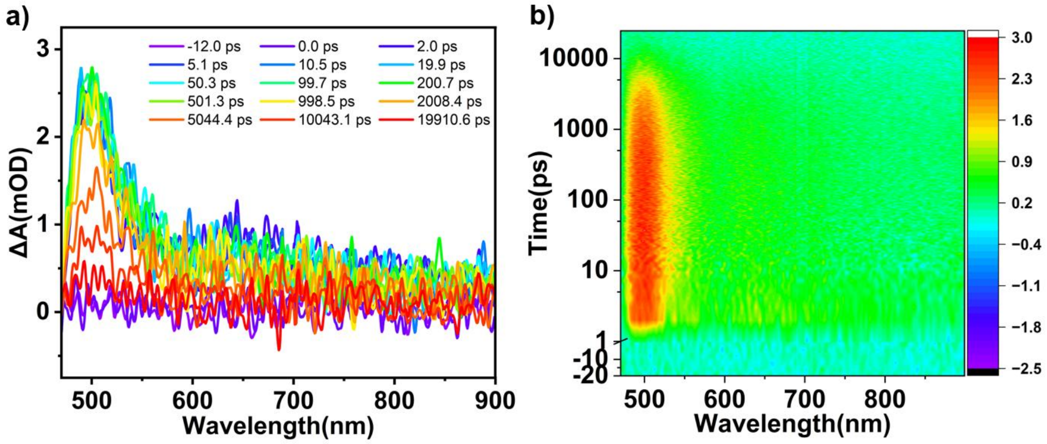

Figure S17. Nanosecond transient absorption spectra of **1**•8Cl^{-} (10 μM) in water. λ_{ex} = 400 nm

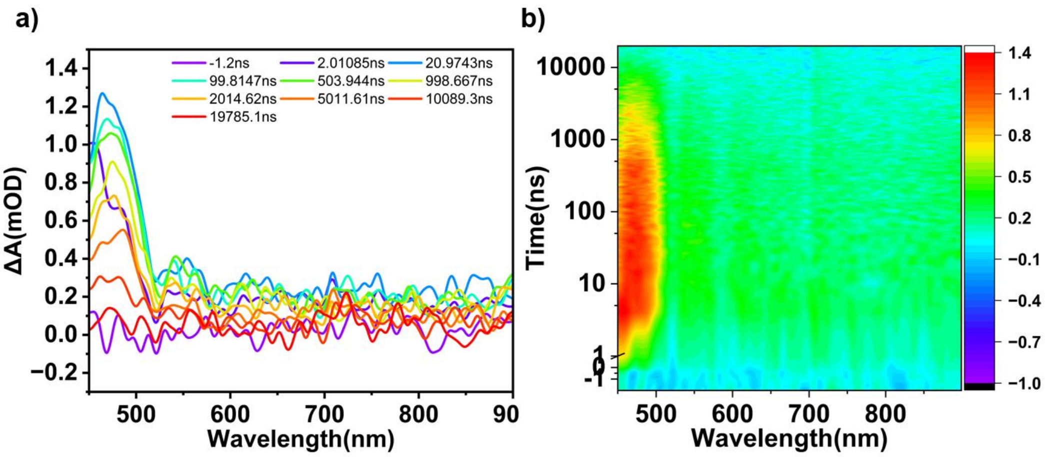

Figure S18. Nanosecond transient absorption spectra of **S3**•4Cl^{-} (200 μM) in water. λ_{ex} = 530 nm

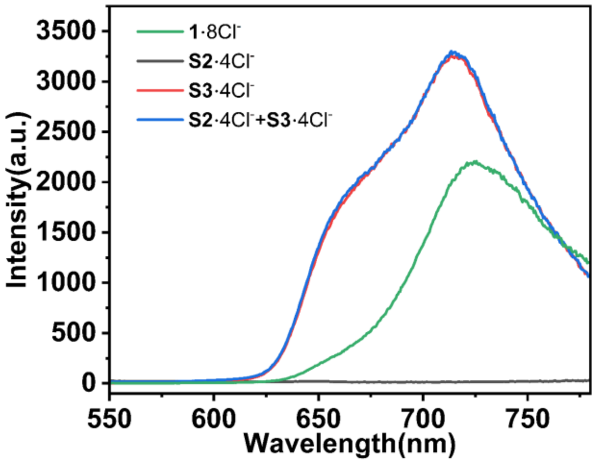

Figure S19. Fluorescence spectra of **1**•8Cl¯ (10 μM), **S2**•4Cl¯ (10 μM), **S3**•4Cl¯ (10 μM) and the 1:1 mixture of **S2**•4Cl¯ (10 μM) and **S3**•4Cl- (10 μM) in water. λ_{ex} = 530 nm.

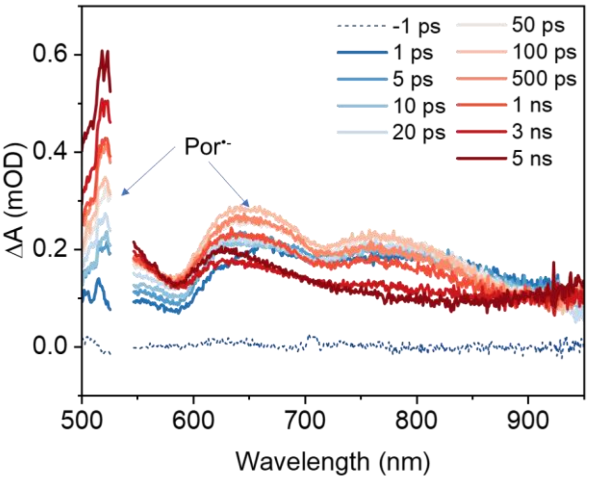

Figure S20. Femtosecond transient absorption spectra of **1**•8Cl¯ (10 μM) in water. λ_{ex} = 530 nm

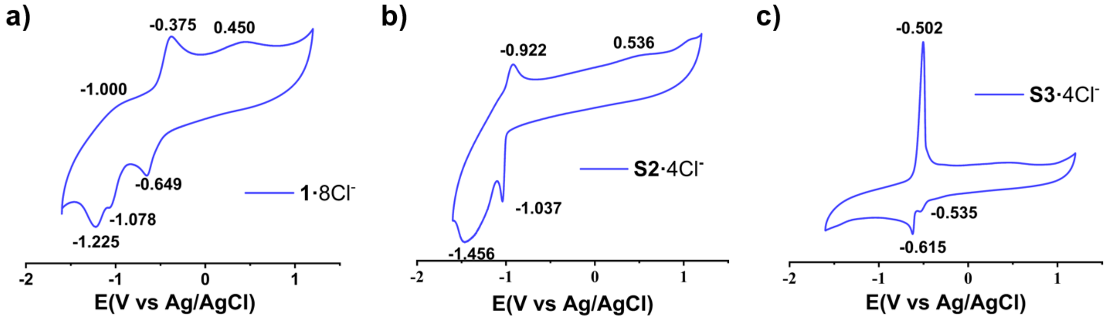

Figure S21. CV data for a) **1**•8Cl¯, b) **S2**•4Cl¯ and c) **S3**•4Cl¯in H_2O.

Table S3. The first reduction and oxidation potential of **1**•8Cl$^-$, **S2**•4Cl$^-$, and **S3**•4Cl$^-$ in H_2O.

Compound	$E_{red,1}$/V	$E_{ox,1}$/V
**1**•8Cl$^-$	-0.649	0.450
**S2**•4Cl$^-$	-1.037	0.536
**S3**•4Cl$^-$	-0.535	–

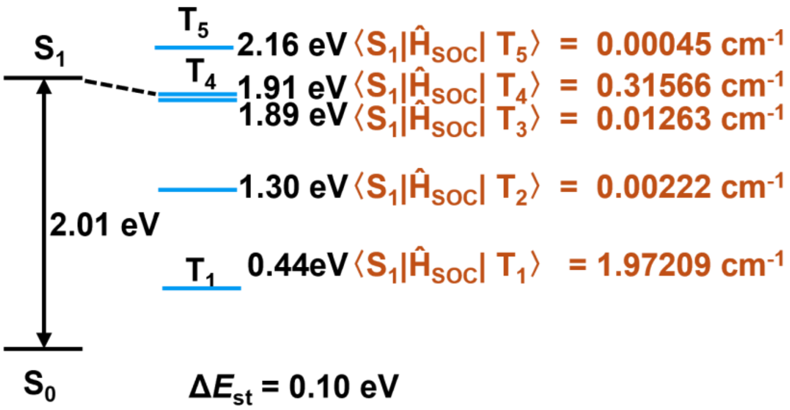

Figure S22. Calculated energy levels of the singlet and triplet states of 1, and spin-orbit coupling matrix elements (SOCMEs), for the S_0 equilibrium structures at the CAM-B3LYP/6-31G* level with DKH2 relativistic corrections.

ROS generation

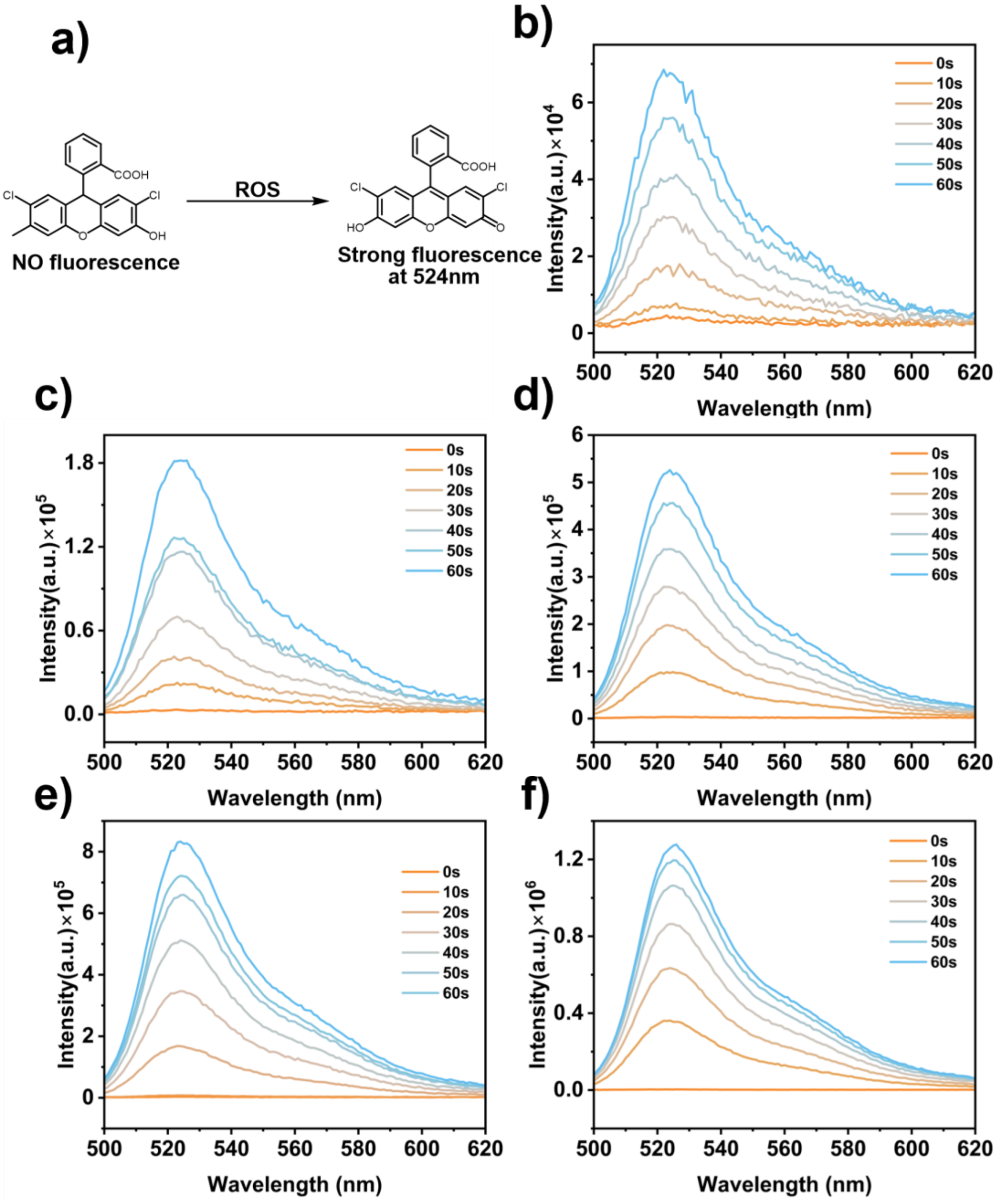

Figure S23. The ROS generation of PSs. (a) The mechanism of 2',7'-dichlorodihydrofluorescein (DCFH) as the scavenger monitors any general types of ROS in the solution. Fluorescence emission spectra of DCFH (5 μM) under white light irradiation (20 mW cm^{-2}) for different time intervals in the following systems: (b) no photosensitizer added; (c) with **S2**•4Cl^{-} (1 μM); (d) with **S3**•4Cl^{-} (1 μM); (e) with a 1:1 mixture of **S2**•4Cl^{-} and **S3**•4Cl^{-} (each at 1 μM); (f) with **1**•8Cl^{-} (1 μM).

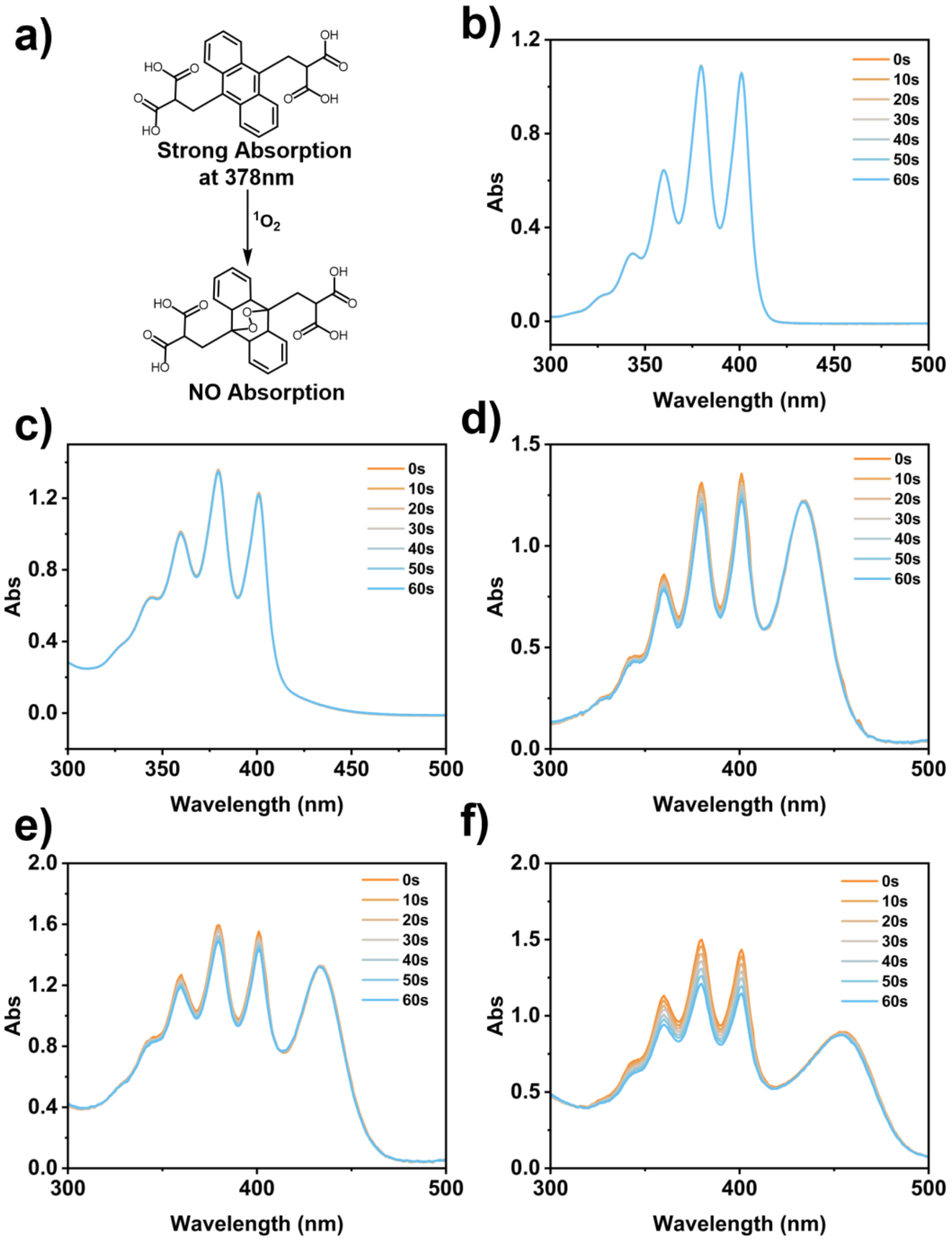

Figure S24. The $^1O_2$ generation of PSs. a) The mechanism of 9,10- anthracenediyl-bis(methylene)-dimalonic acid (ABDA) as the scavenger monitors any general types of $^1O_2$ in the solution. The UV/vis spectra of ABDA (100 μM) under white light irradiation (20 mW cm^{-2}) for different time intervals in the following systems: (b) no photosensitizer added; (c) with **S2**•4Cl^- (10 μM); (d) with **S3**•4Cl^- (10 μM); (e) with a 1:1 mixture of **S2**•4Cl^- and **S3**•4Cl^- (each at 10 μM); (f) with **1**•8Cl^- (10 μM).

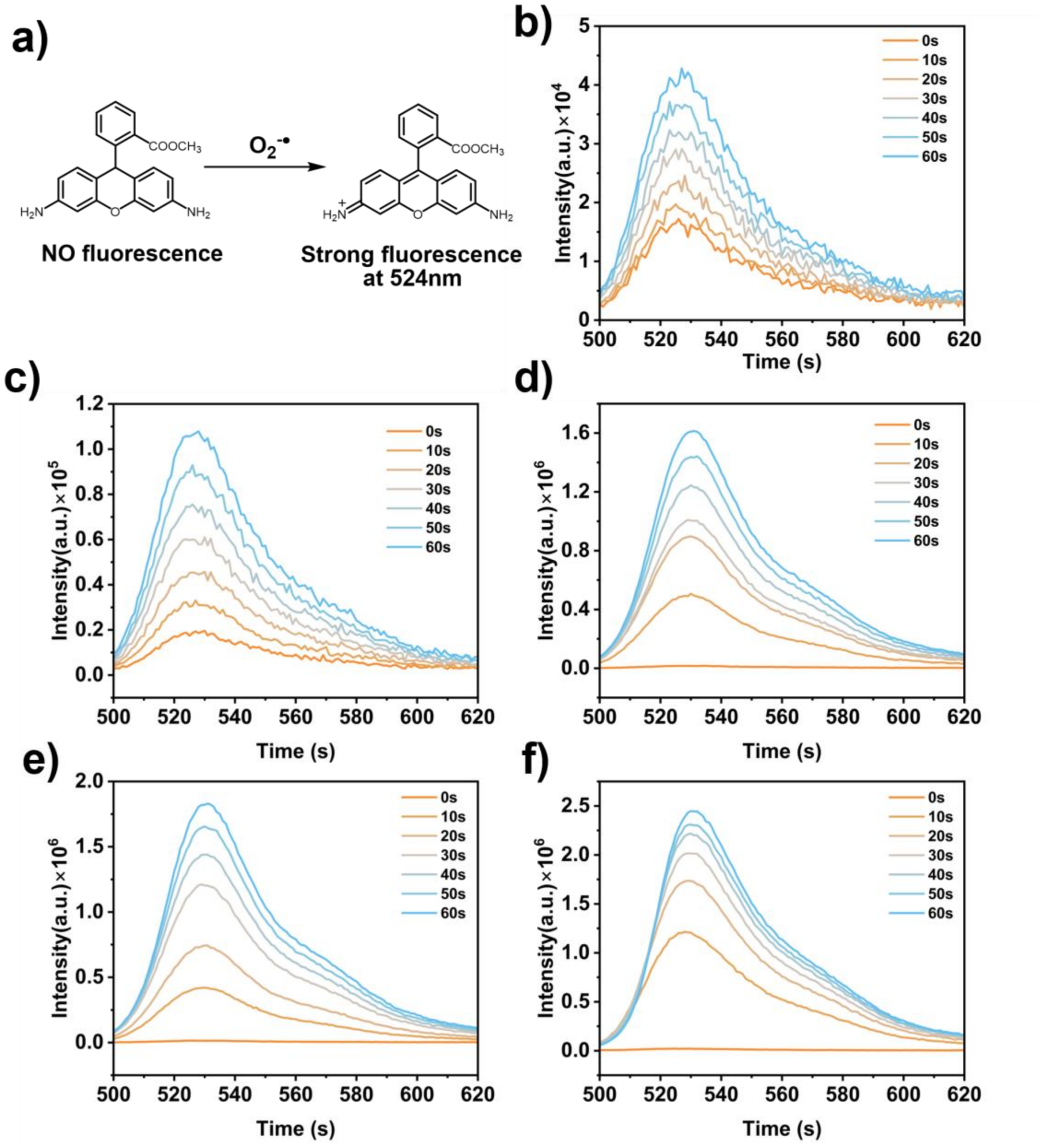

Figure S25. The $O_2^{\bullet-}$ generation of PSs. a) The mechanism of dihydrorhodamine 123 (DHR123) as the scavenger monitors any general types of $O_2^{\bullet-}$ in the solution. Fluorescence emission spectra of DHR123 (60 μM) under white light irradiation (20 mW cm^{-2}) for different time intervals in the following systems: (b) no photosensitizer added; (c) with **S2**•4Cl$^-$ (10 μM); (d) with **S3**•4Cl$^-$ (10 μM); (e) with a 1:1 mixture of **S2**•4Cl$^-$ and **S3**•4Cl$^-$ (each at 10 μM); (f) with **1**•8Cl$^-$ (10 μM).

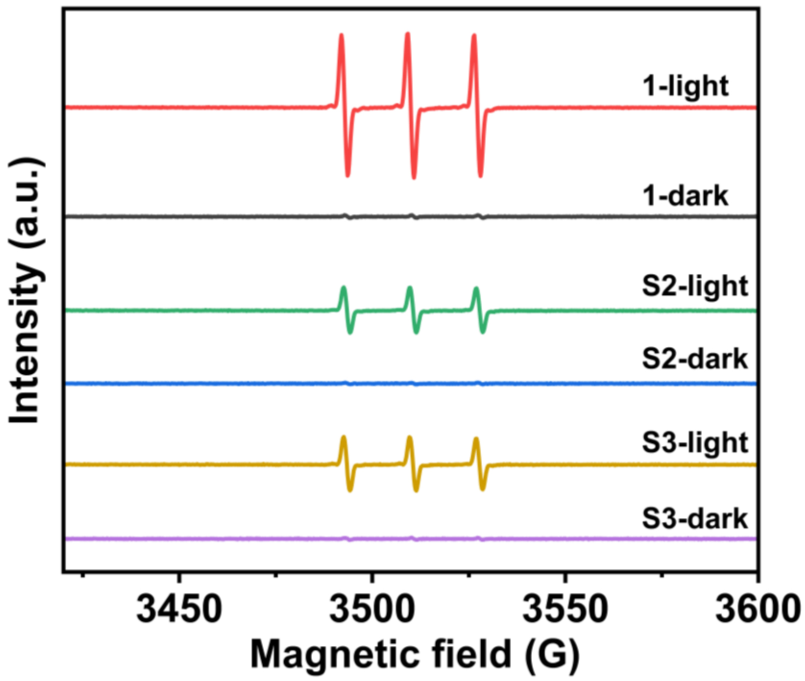

Figure S26. The EPR spectra of $^1O_2$ were detected for **1**•8Cl$^-$, **S2**•4Cl$^-$ and **S3**•4Cl$^-$ (0.1 mM) with/without white light irradiation (Xe lamp, 300 W) for 30 s, using TEMP (100 mM) as spin trap agents.

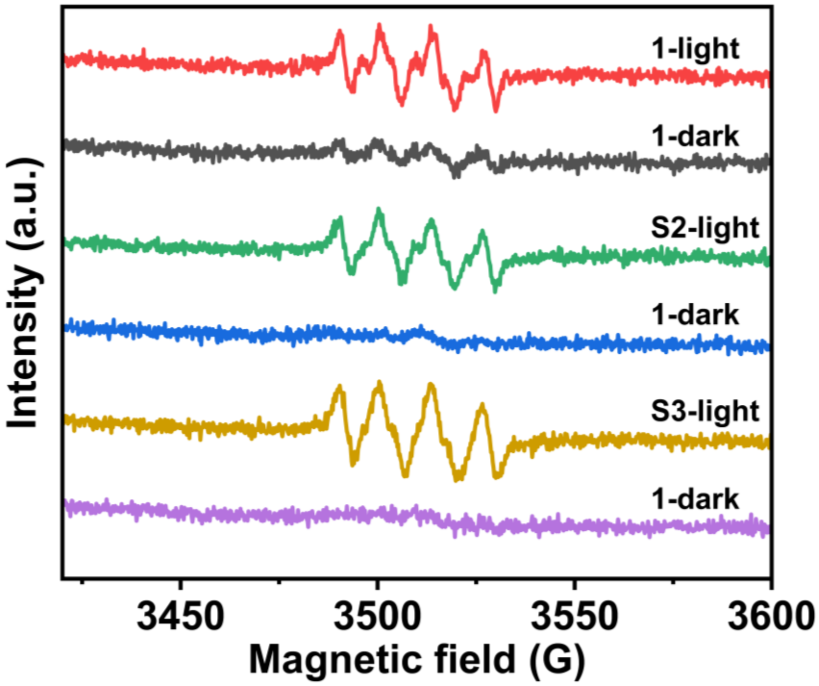

Figure S27. The EPR spectra of $O_2^{-\bullet}$ were detected for **1**•8Cl$^-$, **S2**•4Cl$^-$ and **S3**•4Cl$^-$ (0.1 mM) with/without white light irradiation (Xe lamp, 300 W) for 30 s, using DMPO (250 mM) as spin trap agents.

The host-guest experiments and oxidation reactions of NADH

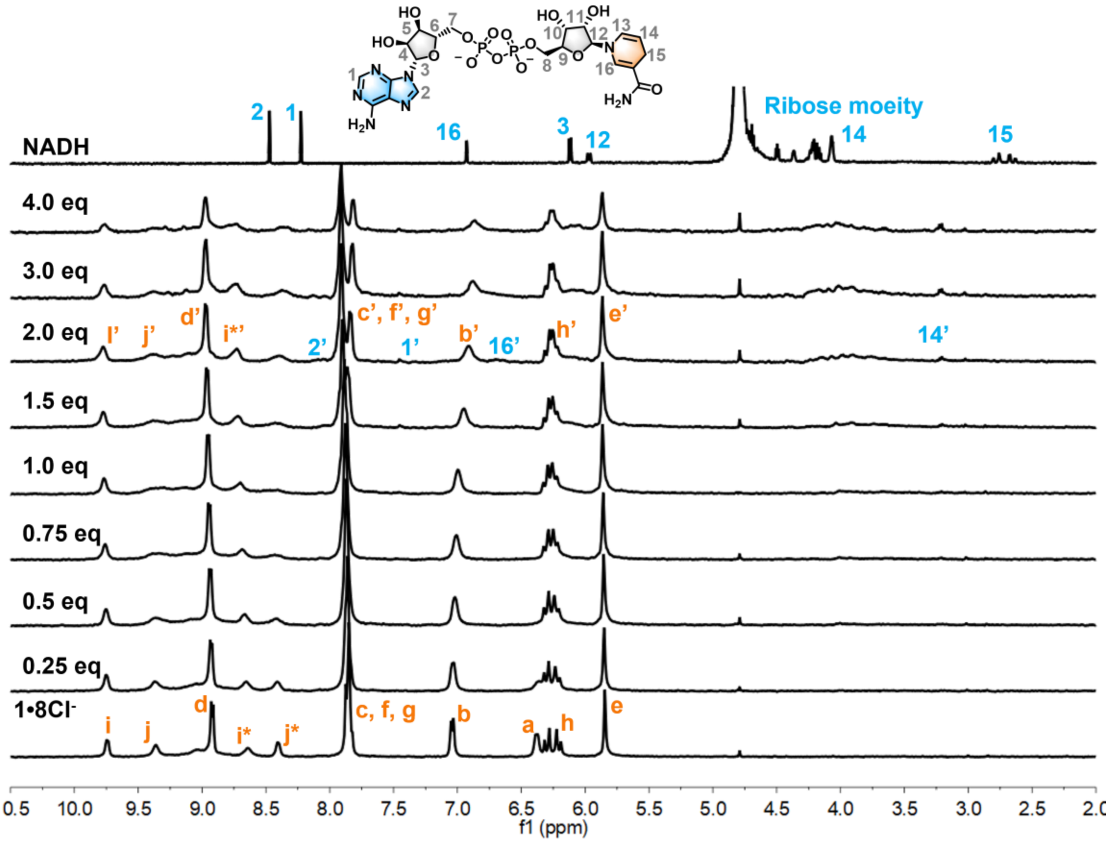

Figure S28. ^{1}H NMR titration (400 MHz, 298 K, D_2O) of **1**•8Cl$^-$ (0.40 mM) with NADH (0 – 4.0 equiv).

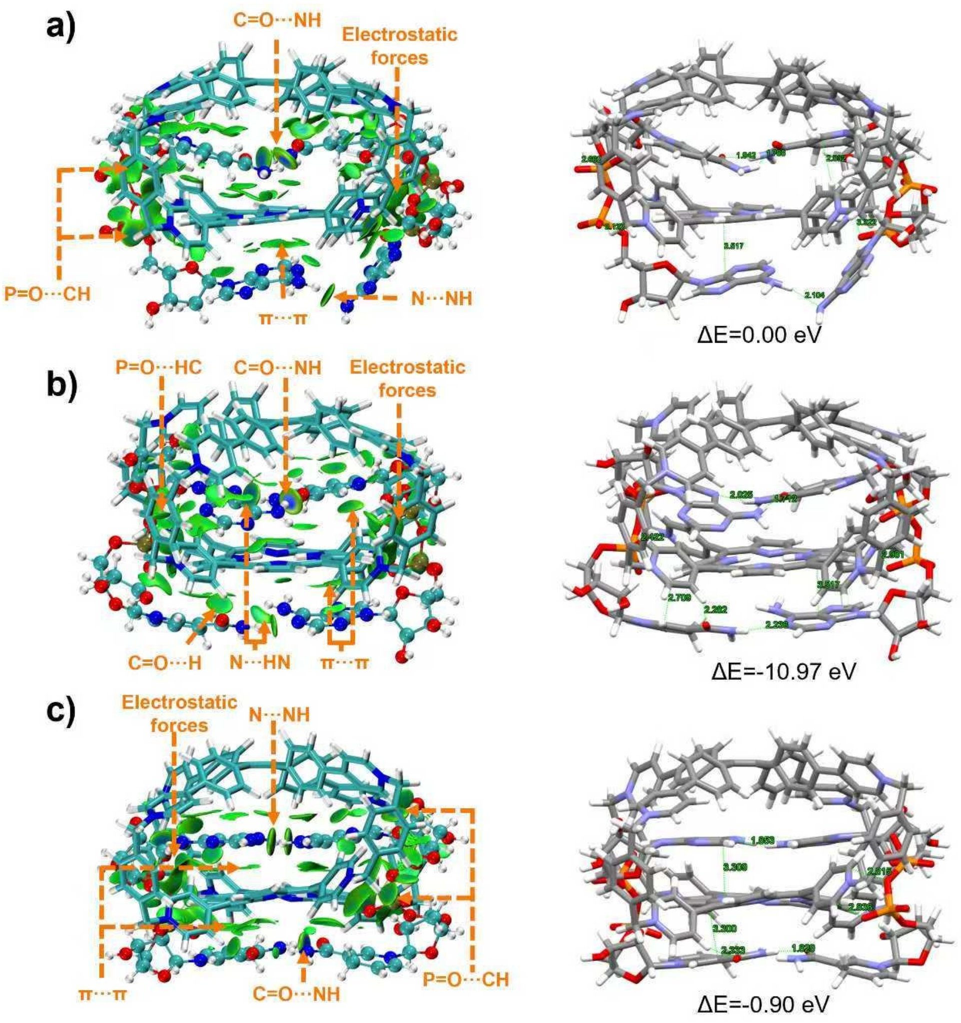

Figure S29. DFT calculations of **1**⊃$NADH_2$. a) Two nicotinamide moieties; b) One adenine and one nicotinamide moiety; c) Two adenine moieties are encapsulated within the cavity of **1**•8Cl^-.

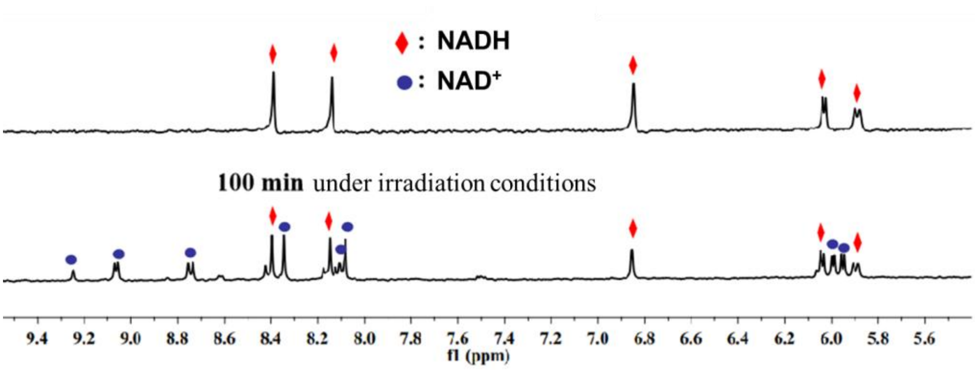

Figure S30. Partial ^{1}H NMR spectra recorded for the oxidation process of NADH (0.3 mM) in the presence of **1**•8Cl^- (15 μM; 5 mol%) upon irradiation (white LED lamp, 20 mW cm^{-2}) in D_2O.

Experimental data *in vitro* and *in vivo*

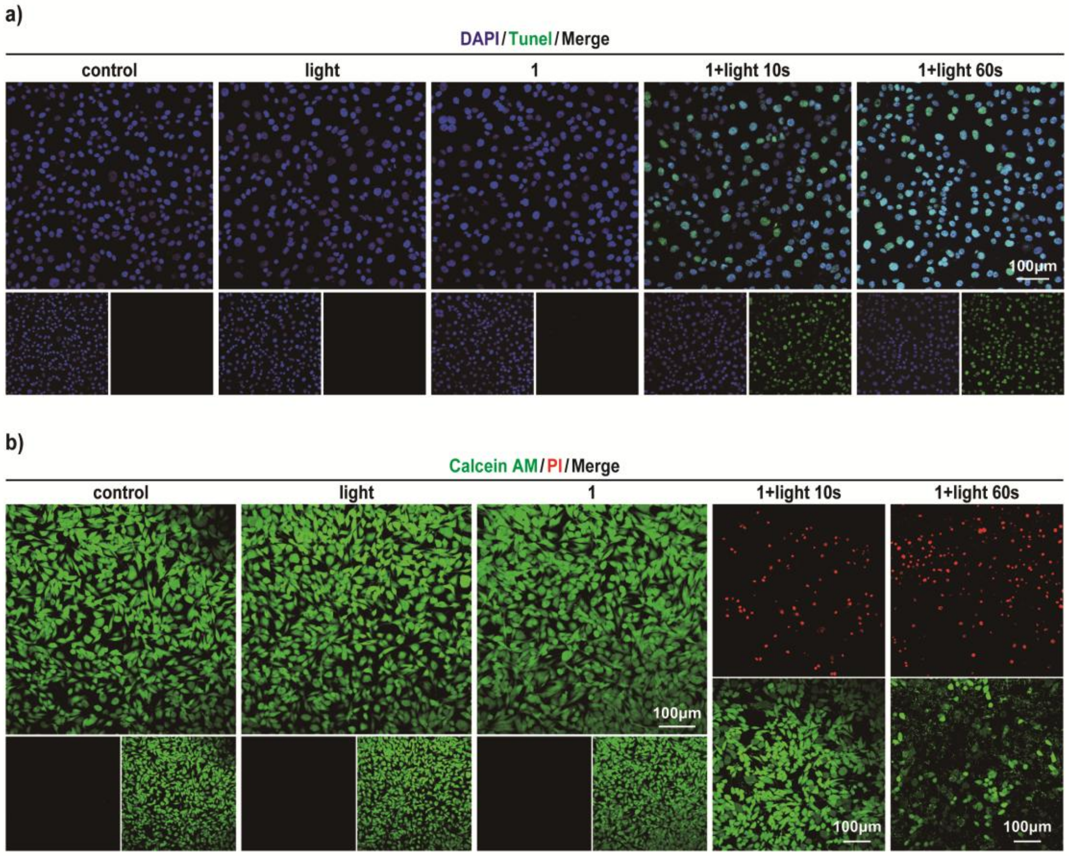

Figure S31. TUNEL staining and Live/Dead assays for various treatments. a) TUNEL staining of cells under different treatment conditions. b) Live/Dead staining of cells under different treatment conditions.

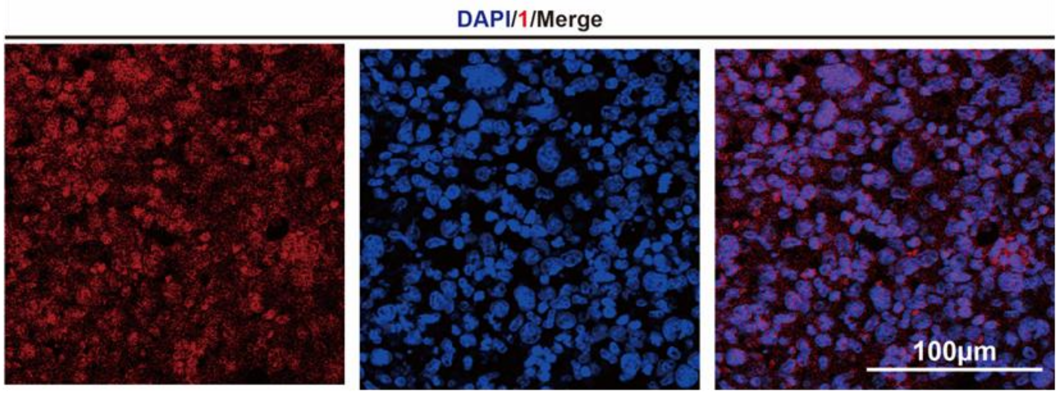

Figure S32. Distribution of **1**•8Cl^{-} within tumor tissue at the cellular level.

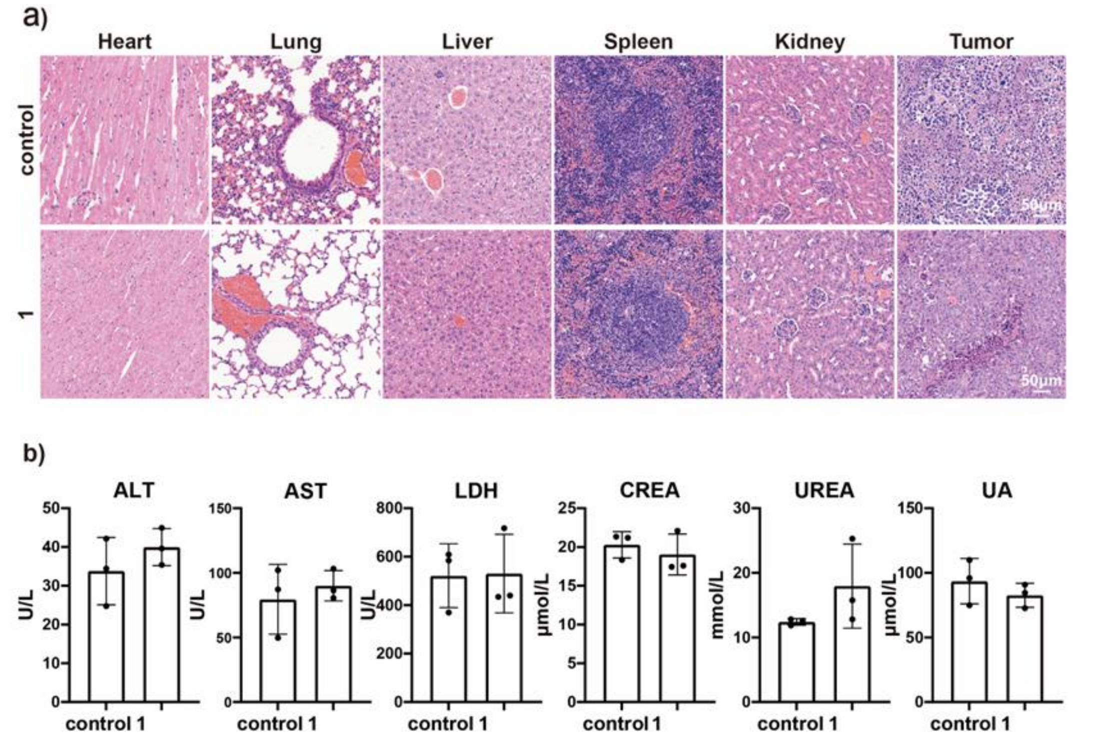

Figure S33. Toxicity of **1**•8Cl⁻ to different organs. a) HE staining of intratumoral injection of **1**•8Cl⁻ on various organs. b) Intratumoral injection of **1**•8Cl⁻ effects on heart, liver, and kidneys.

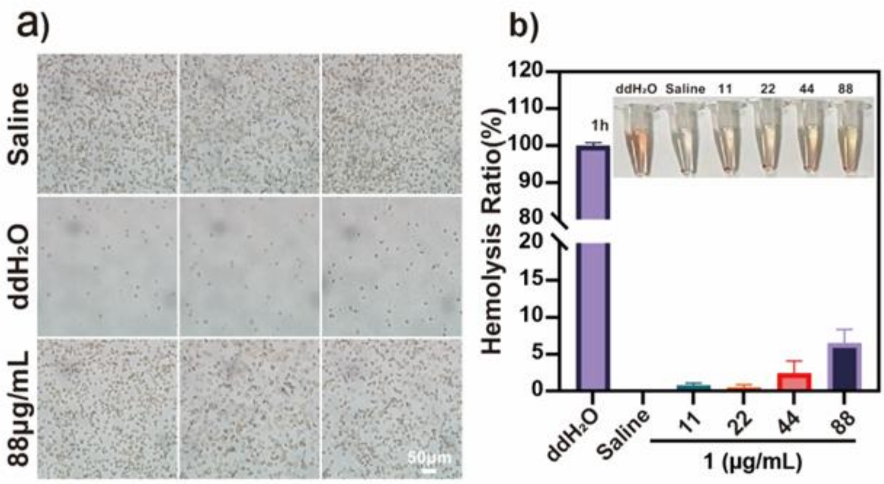

Figure S34. Blood compatibility of **1**•8Cl⁻. a) High concentrations of **1**•8Cl⁻ as well as erythrocyte morphology in the negative and positive control groups. b) Hemolysis rate at different concentrations of **1**•8Cl⁻.

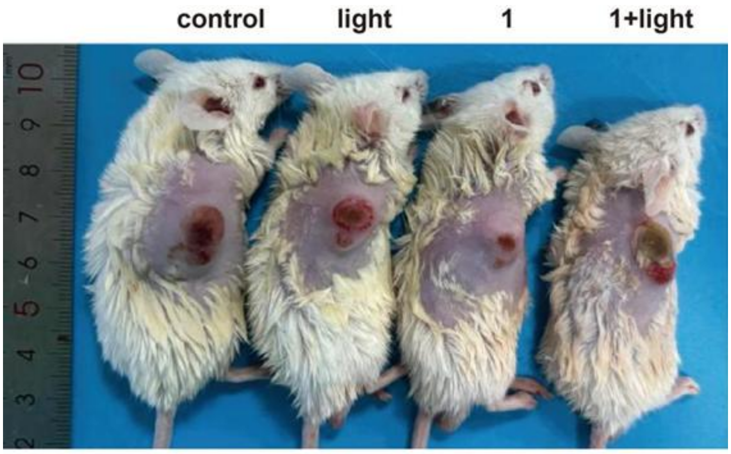

Figure S35. Representative plot of tumor changes in mice after treatment in each group.

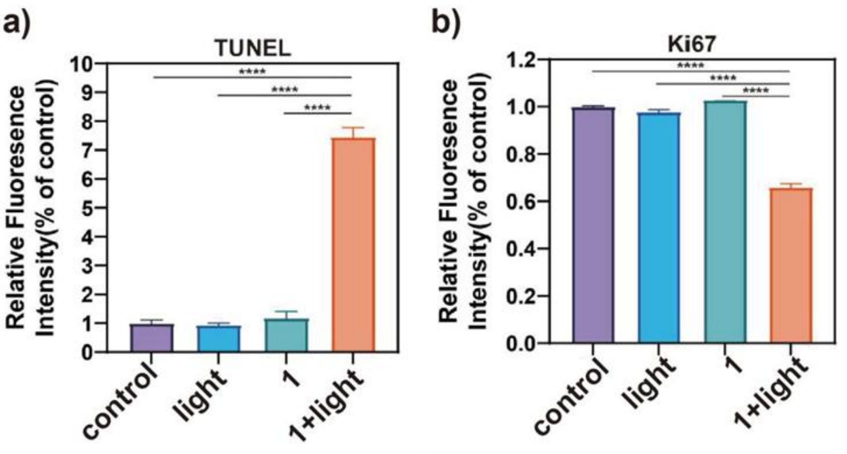

Figure S36. Fluorescence intensity analysis of immunofluorescence in tumor tissues. a) Proportion of TUNEL-positive cells in tumor sections after different treatments. b) Immunofluorescence of Ki67 in tumor sections after treatment in each group. ****, $P < 0.0001$.

References

[1] Duan H, Li Y, Li Q *et al*. Host-Guest Recognition and Fluorescence of a Tetraphenylethene-Based Octacationic Cage. *Angew Chem Int Ed* 2020; **59**: 10101–10.

[2] Li Y, Dong Y, Cheng L *et al*. Aggregation-Induced Emission and Light-Harvesting Function of Tetraphenylethene-Based Tetracationic Dicyclophane. *J Am Chem Soc* 2019; **141**: 8412–5.

[3] Hu H, Wang H, Yang Y *et al*. A Bacteria-Responsive Porphyrin for Adaptable Photodynamic/Photothermal Therapy. *Angew Chem Int Ed* 2022; **61**: e202200799.

[4] Dirac PAM. Note on Exchange Phenomena in the Thomas Atom. *Math Proc Cambridge* 1930; **26**: 376–85.

[5] Vosko SH, Wilk L, Nusair M. Accurate Spin-Dependent Electron Liquid Correlation Energies for Local Spin Density Calculations: A Critical Analysis. *Can J Phys* 1980; **58**: 1200–11.

[6] Becke AD. Density-Functional Exchange-Energy Approximation with Correct Asymptotic Behavior. *Phys Rev A* 1988; **38**: 3098–100.

[7] Lee C, Yang W, Parr RG. Development of the Colle-Salvetti correlation-energy formula into a functional of the electron density. *Phys Rev B* 1988; **37**: 785–9.

[8] Becke AD. Density-Functional Thermochemistry. III. The Role of Exact Exchange. *J Chem Phys* 1993; **98**: 5648–52.

[9] Stephens PJ, Devlin FJ, Chabalowski CF *et al*. Ab Initio Calculation of Vibrational Absorption and Circular Dichroism Spectra Using Density Functional Force Fields. *J Phys Chem* 1994; **98**: 11623–7.

[10] Ditchfield R, Hehre WJ, Pople JA. Self-Consistent Molecular-Orbital Methods. IX. An Extended Gaussian-Type Basis for Molecular-Orbital Studies of Organic Molecules. *J Chem Phys* 1971; **54**: 724–8.

[11] Gao X, Bai S, Fazzi D *et al*. Evaluation of spin-orbit couplings with linear-response time-dependent density functional methods. *J Chem Theory Comput* 2017; **13**: 515–24.

[12] Lu T, Chen Q. Independent Gradient Model based on Hirshfeld Partition: A New Method for Visual Study of Interactions in Chemical Systems. *J Comput Chem* 2022; **43**: 539–55.

[13] Humphrey WF, Dalke A, Schulten K. VMD: Visual Molecular Dynamics. *J Mol Graph Model* 1996; **14**: 27–38.

[14] Lu T, Chen F. Multiwfn: A Multifunctional Wavefunction Analyzer. *J Comput Chem* 2012; **33**: 580–92.
